# Gain of bipolar disorder-related lncRNA *AP1AR-DT* in mice induces depressive and anxiety-like behaviors by reducing Negr1-mediated excitatory synaptic transmission

**DOI:** 10.1186/s12916-024-03725-0

**Published:** 2024-11-18

**Authors:** Shufen Li, Hongyu Ni, Yaping Wang, Xiaohui Wu, Jianqiang Bi, Haiyan Ou, Zhongwei Li, Junjiao Ping, Zhongju Wang, Renhao Chen, Qiong Yang, Meijun Jiang, Liping Cao, Tingyun Jiang, Siqiang Ren, Cunyou Zhao

**Affiliations:** 1 Department of Medical Genetics, Guangdong Technology and Engineering Research Center for Molecular Diagnostics of Human Genetic Diseases, and Guangdong Engineering and Technology Research Center for Genetic Testing, School of Basic Medical Sciences, and Guangdong Mental Health Center, Guangdong Provincial People’s Hospital (Guangdong Academy of Medical Science), Southern Medical University, Guangzhou, China; 2 Present Address: Key Laboratory of Mental Health of the Ministry of Education, Guangdong-Hong Kong-Macao Greater Bay Area Center for Brain Science and Brain-Inspired Intelligence, Guangdong-Hong Kong Joint Laboratory for Psychiatric Disorders, Guangdong Province Key Laboratory of Psychiatric Disorders, and Guangdong Basic Research Center of Excellence for Integrated Traditional and Western Medicine for Qingzhi Diseases, Southern Medical University, Guangzhou, Guangzhou, China; 3 Shenzhen Kangning Hospital, Shenzhen Mental Health Center, Shenzhen, China; 4 The Third People’s Hospital of Zhongshan, Zhongshan, Guangdong China; 5 Department of Psychiatry, The Affiliated Brain Hospital of Guangzhou Medical University (Guangzhou Huiai Hospital), Guangzhou, China; 6 Guangdong Mental Health Center, Guangdong Provincial People’s Hospital (Guangdong Academy of Medical Science), Guangzhou, China; 7 Experimental Education/Administration Center, School of Basic Medical Science, Southern Medical University, Guangzhou, China

**Keywords:** Monozygotic twin, Epigenetics, Phenotypic variations, Bipolar disorder

## Abstract

**Background:**

Bipolar disorder is a complex polygenic disorder that is characterized by recurrent episodes of depression and mania, the heterogeneity of which is likely complicated by epigenetic modifications that remain to be elucidated.

**Methods:**

We performed transcriptomic analysis of peripheral blood RNA from monozygotic (MZ) twins discordant for bipolar disorder to identify disease-associated differentially expressed long noncoding RNAs (DE-lncRNAs), which were further validated in the PsychENCODE brain RNA-seq dataset. We then performed behavioral tests, electrophysiological assays, chromatin immunoprecipitation, and PCR to investigate the function of DE-lncRNAs in the mouse and cell models. Statistical analyses were performed using GraphPad Prism 9.0 or SPSS.

**Results:**

We identified a bipolar disorder-associated upregulated long non-coding RNA (lncRNA), *AP1AR-DT*. We observed that overexpression of *AP1AR-DT* in the mouse medial prefrontal cortex (mPFC) resulted in a reduction of both the total spine density and the spontaneous excitatory postsynaptic current (sEPSC) frequency of mPFC neurons as well as depressive and anxiety-like behaviors. A combination of the results of brain transcriptome analysis of *AP1AR-DT* overexpressing mice brains with the known genes associated with bipolar disorder revealed that *NEGR1*, which encodes neuronal growth regulator 1, is one of the *AP1AR-DT* targets and is reduced in vivo upon gain of *AP1AR-DT* in mice. We further demonstrated that overexpression of recombinant *Negr1* in the mPFC neurons of *AP1AR-DT*_*OE*_ mice ameliorates depressive and anxiety-like behaviors and normalizes the reduced excitatory synaptic transmission induced by the gain of *AP1AR-DT*. We finally identified that *AP1AR-DT* reduces NEGR1 expression by competing for the transcriptional activator NRF1 in the overlapping binding site of the *NEGR1* promoter region.

**Conclusions:**

The epigenetic and pathophysiological mechanism linking *AP1AR-DT* to the modulation of depressive and anxiety-like behaviors and excitatory synaptic function provides etiological implications for bipolar disorder.

**Supplementary Information:**

The online version contains supplementary material available at 10.1186/s12916-024-03725-0.

## Background

Bipolar disorder is a severe, highly heritable mental illness characterized by recurrent episodes of depression and mania over the course of a lifetime [1]. Genetic studies have demonstrated that the lifetime risk of developing bipolar disorder for first-degree relatives of affected individuals is 8.7% [2]. Despite the significant progress made by genome-wide association studies (GWAS) in understanding the genetic architecture, the genetic variants identified are common and have small effect sizes. Together, the implicated variants account for only 25% of the genetic contribution to bipolar disorder. Furthermore, the concordance rate for bipolar disorder in monozygotic twins is approximately 70% [3]. This suggests that there are more complex interactions between genetic and environmental factors that play an essential role in the pathophysiology of bipolar disorder and determine the recurrent episodes. Genetic factors can only explain part of the susceptibility [4]. Environmental risk factors may play an important role in bridging the gap between the genetic basis of bipolar disorder and environmental insults. There is evidence that environmental risk factors, such as irritable bowel syndrome, childhood adversity, obesity, early life stress, and cannabis abuse or dependence, may increase susceptibility to bipolar disorder [5–8]. In recent years, epigenetic modifications, including non-coding RNA, DNA methylation, and other types of modifications, have been proposed to mediate environmental factors and induce susceptibility to bipolar disorder through their involvement in nervous system development, synaptic transmission, immune dysfunction, and metabolic pathway [5, 9–11]. However, precise mechanism remains to be elucidated.

Of the major epigenetic modifications, long non-coding RNA (lncRNA) represents a category of non-protein-coding transcripts with a minimum length of 200 nucleotides. LncRNAs are involved in the transcriptional or post-transcriptional regulation of genes by interacting with DNA, RNA, or proteins via a structural domain formed by complementary base pairing and transcript folding [12]. LncRNAs are believed to play a role in the development and cellular complexity of the human brain, which may contribute to higher levels of cognition and self-awareness [13]. Brain development and maturation continue throughout life, and abnormal development can lead to nervous system disorders such as bipolar disorder, schizophrenia, and major depressive disorder. The function of lncRNA in brain development includes regulation of neurogenesis, synaptic plasticity, and neuronal excitability [14]. For instance, the lncRNA *BC200*, which contains the bipolar disorder-associated SNP rs4404327, is specifically expressed in neurons and has been demonstrated to regulate neuronal dendritic arborization, which directly affects synaptic plasticity and memory [15]. Neuronally expressed *neuroLNC* regulates synaptic vesicle release by selectively stabilizing presynaptic protein-coding mRNA through interaction with the RNA-binding protein TDP-43 (TAR DNA-binding protein-43) [16]. The lncRNA *NEAT1* plays an important role in neuronal excitability by binding to potassium channel interacting proteins [17]. These findings indicate the significant role of lncRNAs in neuronal and synaptic function. A substantial body of clinical evidences indicants that aberrant synaptic and cellular plasticity plays a pivotal role in the pathogenesis of bipolar disorder [18]. Analysis of *Nissl-*stained sections of the left dorsolateral prefrontal cortex (DLPFC) from post-mortem brain tissue in bipolar disorder and major depression revealed significant reductions in neuronal density and cell size in specific layers, which correlated with less extensive or less active axodendritic trees[19–22]. A study of iPSC-derived brain organoids generated from monozygotic (MZ) twins discordant for psychiatric disorder demonstrated that a shift in the balance of neuronal and synaptic prevalence towards GABAergic specification during early cortical development is one of the endophenotypes of psychosis [23].

In this study, we performed RNA sequencing of peripheral blood RNA obtained from bipolar disorder discordant (BDC) MZ twins and healthy concordant control (HCC) MZ twins to screen for bipolar disorder-associated differentially expressed lncRNAs (DE-lncRNAs). We then validated the function of the DE-lncRNA *AP1AR-DT* in a mouse model and revealed the regulatory molecular mechanism by which the overexpression of *AP1AR-DT* in mice induces depressive and anxiety-like behaviors by reducing Negr1-mediated excitatory synaptic transmission. This finding links *AP1AR-DT* to the behavioral and pathophysiological abnormalities and provides an etiological implication for bipolar disorder.

## Methods

### Subjects

Peripheral blood samples were obtained from 5 pairs of BDC twins and 4 pairs of healthy concordant control (HCC) twins. RNA extracted form blood samples was subjected to RNA-seq analysis (Additional file 1: Table S1). All patients met the diagnostic criteria for bipolar disorder according to the Diagnostic and Statistical Manual of Mental Disorders, 4th Edition (American Psychiatric Association). All participants in this study provided informed consent prior to the study following presentation of the nature of the procedures. The study was approved by the Medical Ethics Committee of Zhujiang Hospital of Southern Medical University (#2022-KY-086) and the third People’s Hospital of Zhongshan (SSYLL20210301). The study was conducted in accordance with the Declaration of Helsinki.

### RNA-seq analysis of RNA from MZ twin subjects

RNA extracted from peripheral white blood cells using TRIzol reagent (Invitrogen, Carlsbad, CA, USA) with a minimum RNA integrity value of 7 was used to build a strand-specific RNA library after removal of ribosomal RNA. The library was sequenced to a depth of ~ 100 million 125-bp paired-end reads per sample on an Illumina HiSeq 2000 by Novogene Solution (Tianjin, China). After quality control using FastQC, the reads were aligned to the University of California, Santa Cruz (UCSC) Homo sapiens reference genome hg19 with TopHat version 2.0.4, and multi-aligned reads were removed with SAMtools. We then used HTSeq to generate mapping counts for the known annotated genes, including coding genes for mRNAs and noncoding genes for lncRNAs from the UCSC Homo sapiens hg19 GTF file (version 1.05). We used Cuffdiff to convert the counts into total fragments per kilobase of transcript per million mapped reads (FPKM) values, which represented the gene expression levels. Fold change (FC) in gene expression is computed as the FPKM ratios of the patients over the controls.

Among those lncRNAs, only genes with read counts greater than 0 in 50% of the samples and greater than 5 in 10% of the samples were retained for DE-lncRNA analysis. Among those mRNAs, only genes with read counts greater than 0 in 50% of the samples and greater than 10 in 25% of the samples were retained for DE-mRNA analysis. Differentially expressed (DE) genes between patients with bipolar disorder and nonpsychiatric controls were identified with the Digital Gene Expression used edgeR package in R version 4.1.2 [24], and the read counts for each annotated gene were fit to a negative binomial generalized log-linear model in edgeR. We included a family ID for each of 9 twin pairs to generate a paired design within the same twin-pair in a case–control analysis of 5 pairs of BDC twins (5 bipolar disorder cases vs. 5 healthy controls, 5 vs. 5; |Log_2_FC|> 0.585 and *P* < 0.05) and then validated the results in 9 pairs of twins including 5 BDC twins and 4 HCC twins (5 bipolar disorder cases vs. 13 healthy controls, 5 vs. 13; |Log_2_FC|> 0.585 and *P* < 0.05; Additional file 1: Table S2). Subsequently, the DE-lncRNAs from twins were validated using an independent PsychENCODE brain RNA-seq dataset [25]. Linear correlation and regression analyses between each protein-coding gene and each of the DE-lncRNAs were performed in an iterative fashion. The mRNA that correlated with at least one of the four lncRNAs (Pearson *p* < 0.05 and |*r*|> 0.5) was identified as significantly correlated.

### RNA-seq analysis of RNA from the SK-N-SH cells and mice brain tissues

RNA extracted from the SK-N-SH cell lines was employed for RNA-seq analysis on the Illumina Nova-PE150 by Novogene Solution (Tianjin, China) with a depth of ~ 30 million 150-bp paired-end reads per sample. The raw reads were subjected to quality control with FASTQC, and then clean reads were aligned to the hg19 reference genome using HISAT2 with default parameters and converted the sam file to the bam file (binary format) using samtools. Read counts were derived from the GTF file generated by Stringtie with the parameters -A, -e and using the Python scripts provided by Stringtie. Differentially expressed genes (DEGs) were generated by DESeq2 in R. Gene Ontology (GO) analysis of significant genes were enriched using ToppGene online database (https://toppgene.cchmc.org/enrichment.jsp)) and visualized using the ggplot2 package in R.

RNA extracted from mouse cortical tissues using TRIzol reagent (Invitrogen) with a minimum RNA integrity value of 7 was used to build an RNA library as previously described [26]. Briefly, the library was sequenced to a depth of ~ 22 million 150-bp paired-end reads per sample on an Illumina HiSeq 6000 platform by Novogene Solution. The raw reads were subjected to quality control with FastQC, and clean reads generated from the raw reads were mapped to the GRCm38 mouse assembly in the Ensembl database by GENCODE using Bowtie2 with the default parameters. We used SANtools to convert the sam file to the bam file (binary format). The quantitative files were generated by StringTie using the -A and -e parameters and the GENCODE GRCm38 GTF reference file. We used the Python scripts provided by StringTie to extract the mapping read counts. Differentially expression analysis was performed using the DESeq2 packages, which fitted the analysis of read counts using a negative binomial generalized log-linear model.

### Cell culture, plasmid constructs, reporter assay, qPCR, and Western blotting

The human embryonic kidney (HEK) 293 T cell lines were cultured in Dulbecco’s Modified Eagle’s Medium (DMEM, Life Technologies, USA), while the human neuroblastoma SK-N-SH cell line was cultured in Minimal Essential Medium (MEM, Life Technologies, USA). The medium was supplemented with 10% non-essential amino acids (NEAA, Life Technologies, USA) and alanyl-glutamine (GlutaMAX™, Life Technologies, USA) and maintained at 37 °C with 5% CO_2_.

The full-length lncRNA-*AP1AR-DT* cDNA (ENST00000562919.1) was amplified from peripheral blood RNA and cloned into the TK-pCDH-copGRP-T2A-Puro expression vector (pCDH-*AP1AR-DT*) via the EcoRI and BamHI restriction sites. The human *NRF1* cDNA (ENST00000393232.6) was amplified from peripheral blood RNA and cloned into the pcDNA3.1 ( +) expression vector (pcDNA3.1-*NRF1*) via the NheI and XhoI restriction sites. In order to confirm that *AP1AR-DT* regulates the expression of *NEGR1* through the predicted region of the promoter, a 257-bp fragment of the *NEGR1* proximal promoter region, spanning from 1149 to 1246 bp upstream of the *NEGR1* translational start site, were amplified from human genomic DNA and cloned into a pGL4.18 dual luciferase vector (Promega, Madison, WI, USA) via the XhoI and HindIII restriction sites (pGL-*NEGR1*). The dual luciferase activity was quantified using a Wallac Victor V 1420 Multilabel Counter (PerkinElmer, San Jose, CA, USA) 48 h after the transfection of HEK293T cells with the pGL4.18 dual luciferase reporter construct, which included a Renilla luciferase gene and a control pRL-TK plasmid (Renilla luciferase reporter plasmid, Promega, Madison, WI, USA). All luciferase readings were obtained from more than three individual biological replicates.

Total RNA was extracted from cell lines, mouse brains, and human peripheral blood using TRIzol reagent (Life Technologies). Reverse transcription was then performed using a PrimeScript RT Reagent Kit with gDNA Eraser (Takara, China) to generate complementary DNA (cDNA). A comparative quantitative polymerase chain reaction (qPCR) assay was performed using a LightCycler 96 System (Roche) with SYBR green dye-containing SuperArray PCR master mix (YEASEN, China) and beta-actin and/or *Gapdh* as reference genes. The estimated values were expressed as 2-ΔΔCt values and subjected to statistical analyses. All primers were synthesized by Invitrogen (Additional file 1: Table S3). Western blotting was conducted using rabbit anti-NEGR1 (1:1000, ab133676, Abcam, USA) and mouse-anti NRF1 (1:1000, ab55744, Abcam, USA) antibodies.

### Animals

All mice used were from the C57BL/6 J genetic background. Animal experiments were in full compliance with the National Institutes of Health Guide for Care and Use of Laboratory Animals and were approved by the Institutional Animal Care and Use Committee at Southern Medical University. The environmental conditions were kept constant: food and water ad libitum, 21° ± 0.5 °C, 60 ± 10% relative humidity, 12-h light/dark cycles, and three to five mice per cage as previously described. This study used male animals in all experiments.

### Mouse behavior tests

A recombinant adeno-associated virus (rAAV) carrying lncRNA-*AP1AR-DT* (*AP1AR-DT*_*OE*_) was constructed by cloning the cDNA sequence of *AP1AR-DT* (ENST00000562919.1) into the rAAV-hsyn-CMV-EGFP-WPRE-hGH-pA expression vector via the SacI and BamHI restriction sites. The EGFP fluorescent reporter gene driven by the CMV promoter, while the lncRNA-*AP1AR-DT* driven by the human synapsin I promoter (hysn). A rAAV carrying *Negr1* (AAV-*Negr1*) driven by the human synapsin promoter was also constructed by cloning the cDNA sequence of *Negr1* (ENSMUST00000074015.11) into the rAAV-hsyn-mCherry-WPREs AAV expression vector via the SpeI and BamHI restriction sites. In this recombinant AAV, the *Negr1* and mCherry fluorescent reporter genes had been fused to form a single polypeptide chain. An empty AAV vector without an insert was used as a negative control. The package and purification of the rAAV were conducted in accordance with the instructions provided by the manufacturer (BrainVTA, Wuhan, China). AAV was injected into the mPFC region of 8-week-old C57BL/6 male mice, and mouse behavior tests were conducted 3 weeks after the injection. The order of the mouse behavior experiments was as follows: open field test, three-chamber test, sucrose preference test, Barnes maze, elevated plus maze, tail suspension test, forced swim test. The mice were given a week to rest after each experiment.

The open-field test (OFT) was conducted in an experimental room and the mice were allowed to habituate for 60 min. Each mouse was placed gently in the center of the open-field arena (50 × 50 × 50 cm). Spontaneous behavior of the mice in the arena was recorded for 1 h using a CCD camera mounted on the ceiling. The total distance travelled and the time spent in the central area was analyzed using tracking software [27].

The three-chamber test (TCT) was carried out in a rectangular box with three chambers. The chambers had small square openings (5 cm × 5 cm) between them. The size of each chamber was 20 cm × 40 cm × 22 cm. The outer walls of the chamber were opaque, while the inner partitions were made of clear Plexiglas. Following a 10-min period of acclimation within the chamber, the test mouse was introduced to the center chamber, with the partitions in place. A wire mesh cup containing a novel mouse (S1) was positioned on one side of the chamber, while an empty mesh cup (E) was placed on the other side during the socialization session. Subsequently, the partitions were elevated, thereby enabling the test mouse to roam freely throughout the chamber. Following a 10-min interval, the test mouse was relocated to the center area, which was devoid of any obstructions. A second novel mouse (S2) was placed under the empty cup, the partitions were removed, and the test mouse was permitted to roam freely throughout the chamber for a period of 10 min during the social novelty preference session. The time spent in each of the three chambers during each test session was quantified using LabMaze V3.0.

For the sucrose preference test, mice were housed in individual cages. The mice were habituated to a 1% sucrose solution for 4 days as follows: on days 1 and 2, bottles A and B were filled with water, and on days 3 and 4, bottles A and B were filled with 1% sucrose solution. The sucrose preference test was performed between days 5 and 7. During the test days, bottle A contained 1% sucrose solution and bottle B contained water. Fluid consumption from each bottle was measured daily. The position of the bottles was changed every day. The remaining volume in each bottle was measured at the end of each day. We calculated sucrose preference as the ratio of sucrose intake to total fluid intake and expressed the values as percentages.

The Barnes maze test was conducted in a circular maze (92 cm in diameter) with 20 small holes (5 cm in diameter) 3 cm from the perimeter on a customized stand (105 cm high). The maze, comprising four distinct cues, was divided into four quadrants theoretically. One of these quadrants was selected as the target quadrant. The day before training, the mouse was placed in the center of the platform with light (400 lx) as an aversive stimulus, allowed to move freely for 10 s to familiarize itself, and gently led to the target box where it rested for 2 min. Training sessions continued for 4 consecutive days. In the training phase, if the mouse entered the target box in 5 min free explore after the bucket was removed, it was allowed to rest for 60 s and was gently returned to the home cage; otherwise, it was gently guided to the hole containing the target box and rested for 60 s. The day after the last training session, a probe trial was performed to assess spatial reference memory. After the target box was removed, the test mouse was placed in the center of the maze as in the training trials and allowed to search for the target quadrant. The time spent in each quadrant was analyzed by the EthoVision system [28].

The elevated plus maze (EPM) was employed to assess anxiety-like behavior. The behavioral apparatus comprised two open arms (width 5 cm × length 30 cm) and two closed arms (width 5 cm × length 30 cm), elevated 50 cm above the floor and illuminated at a low intensity. Mice were placed individually in the center of the maze, facing an open arm, and permitted to freely explore for 5 min. For a mouse to be considered to have entered an arm, all four paws had to be inside the entrance line. The time spent in each arm and the end time were determined when all four paws were back to the line again. The maze was cleaned with 70% ethanol after each test to prevent influence from the previously tested mouse. The time spent in the open and closed arms was analyzed using video tracking software.

For the tail suspension test (TST), each animal was suspended by its tail via a small aluminum plate that was attached to the tail approximately 1 cm from the base with adhesive medical tape; the plate was hooked to an attachment located inside an opaque box (25 × 25 × 50 cm). The behaviors of the mice were recorded for 5 min using a charge-coupled device (CCD) camera, which was mounted inside the box. The experiments were recorded and immobility time scored by the same experimenters, who were unaware of the animals’ genotypes [29].

The forced swim test (FST) was conducted for a duration of five minutes for the primary test. Prior to the main test, a 24-h pretest was performed by placing each experimental mouse in a transparent cylinder filled with water (23–25 °C; depth 15 cm) for a period of 15 min. The immobility observed in the FST was defined as a state in which the mice were judged to be making only the movements necessary to keep their head above the surface. The experiments were video recorded and scored by the same experimenters, who were unaware of the animals’ genotypes [30].

### Immunofluorescence analysis, Golgi staining, and image analysis

The whole brain was first perfused with saline and 4% paraformaldehyde and then was fixed in a 4% paraformaldehyde at 4 °C and dehydrated with 30% sucrose until the brain sinks to the bottom of the tube. After preparation of 40 μm coronal sections with a Leica CM1950 cryostat, transfer them onto slides. Fluorescent images were obtained with a confocal microscope (LSM880, Carl Zeiss, Jena, Germany).

Golgi staining using a kit from FD NeuroTechnologies was performed as previously described. Briefly, brains from five pairs of independent *AP1AR-DT*_*OE*_ mice, control mice, or *Negr1-*RE mice were harvested whole and rinsed with distilled water. The brains were incubated in a 1:1 FD solution A: B mixture for 24 h at room temperature in darkness. Subsequently, the brains were transferred to fresh solution mixture of FD solution A: B for 2 weeks at room temperature in darkness. The brains were then transferred into FD solution C and stored in darkness at room temperature for 48 h. Coronal Sects. (150 μm) were cut with a Leica CM1950 cryostat and mounted on gelatin-coated slides coated with 3% gelatine. Slides were dehydrated in ethanol and mounted in neutral resin according to the manufacturer’s protocol. The dendrites were traced using the Fiji plugin NeuronJ. The spine densities were quantified by tracing the dendritic segments, with the spines counted in the mPFC region [31].

Z-stack images were obtained at 1-μm intervals for Sholl analysis or 0.3-μm intervals for spine analysis) using a BX63 microscope (Olympus, Japan) with a 40 × or 100 × oil immersion lens. Image analyses and quantification were conducted using the Fiji software. For photo-micrographic illustration of dendritic spines, the images were processed with CellSens Dimension software (Olympus) to deblur the images caused by spines oriented in different depths of focal planes and pyramidal neurons.

### sEPSCs and PPRs analyses

Mice were deeply anesthetized with isoflurane and perfused with ice-cold artificial cerebrospinal fluid (ACSF). Brains were removed and sliced in cutting buffer (containing (mM): 26 NaHCO3, 1.3 NaH2PO4, 12 MgSO4, 0.2 CaCl2, 2.5 KCl, 10 D-glucose and 195 sucrose saturated with 95% O2/5% CO2). The sections were transferred to recording buffer (containing (mM): 126 NaCl, 2.5 KCl, 1.25 NaH2PO4, 1 MgSo4, 2 CaCl2, 26 NaHCO3 and 10 glucose) and allowed to recover for 30 min. Whole-cell patch-clamp recordings were performed using a Multi-Clamp 700B amplifier, with signals being recorded/analyzed using a Digidata 1550B data acquisition system and the pClamp 10. software package (Molecular Devices). Recordings were made from visually identified pyramidal neurons of the mPFC region using patch pipettes (4 to 6 MΩ) when filled with intracellular solutions (containing (mM): 126 mM K-gluconate, 2.5 mM KCl, 10 mM Hepes, 2 mM MgCl_2_, 0.2 mM EGTA, 4 mM Na_2_-ATP, and 0.4 mM Na_2_-GTP). sEPSCs were recorded with pipette solution at a holding potential of − 70 mV in a voltage-clamp mode. Paired postsynaptic currents were elicited by pairs of identical stimuli separated by intervals of 25, 50, 100, and 200 ms with a bipolar -stimulating electrode positioned in the local area of mPFC.

### Chromatin isolation by RNA purification (ChIRP)

We performed ChIRP to verify the interaction of lncRNA-*AP1AR-DT* with the *NEGR1* promoter region using a Chromatin Isolation by RNA Purification (ChIRP) Kit (BersinBio). Briefly, HEK293T cell lysates were cross-linked with 1% formaldehyde 10 min after transfection with pCDH-*AP1AR-DT* and then sonicated using a Qsonica instrument (USA). A total of 6 biotinylated tilling probes were employed to capture *AP1AR-DT* by hybridization with the sonicated chromatin in two separate reactions: one for odd-number probes (#1, 3, 5) and another for even-numbered probes (#2, 4, 6) synthesized by RiboBio technologies (RiboBio). The chromatin complexes associated with *AP1AR-DT* were pulled down using streptavidin-conjugated magnetic beads and used for RNA and bound DNA isolation. The levels of bound *NEGR1* promoter from isolated DNA were quantified by qPCR using primers spanning the *AP1AR-DT* binding site adjacent region (Additional file 1: Table S3) followed by sequencing of the qPCR products.

### Chromatin immunoprecipitation (ChIP)

ChIP assays were performed on cell extracts from SK-N-SH cells using anti-NRF1 antibody (ab5574422, Abcam) as recommended (EZ-ChIP, Merck, Germany). Cell lysates extracted from SK-N-SH cells infected with pCDH-*AP1AR-DT* or an empty control vector were subjected to ChIP assays using NRF1 antibody 48 h after transfection with pcDNA3.1-*NRF1*, and the immunoprecipitated DNA was quantified by qPCR using primers partially overlapping the *AP1AR-DT* binding region to evaluate the interaction of NRF1 with the *NEGR1* promoter region. We used Input as a positive control and lgG as a negative control.

### Statistical analysis

Statistical analyses were conducted using GraphPad Prism 9.0 or SPSS, including Pearson correlation analysis, two-tailed Student’s *t* test, and two-way ANOVA test. Data are presented as the mean ± standard error (SE). The level of significance was set at *P* < 0.05.

## Results

### Identification of bipolar disorder-associated DE-lncRNAs within BDC twins

**Fig. 1:**
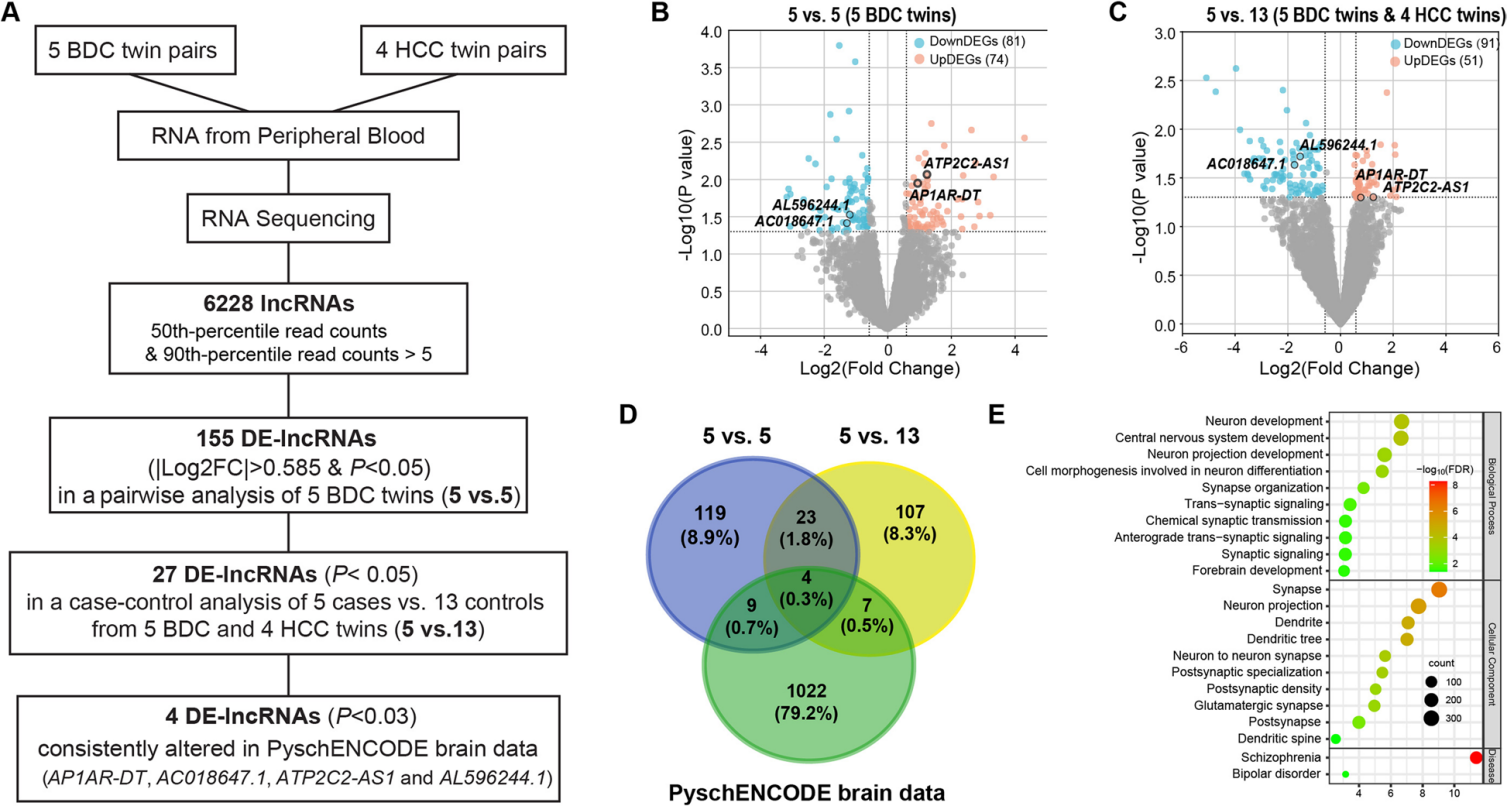
Screening of bipolar disorder-associated DE-lncRNAs. **A** Flowchart for the screening of the bipolar disorder-associated DE-lncRNAs. **B**, **C** Volcano plots showing raw p values versus fold changes for lncRNAs identified in a pairwise analysis of 5 bipolar disorder vs. 5 healthy controls (5 BDC twins for 5 vs. 5; **B**) or in a case–control analysis of 5 bipolar disorder (from 5 BDC twins) vs. 13 healthy controls (by including 4 HCC twins, 5 vs. 13; **C**), with red/blue spots denoting lncRNA with |Log_2_FC|> 0.585 and *P* < 0.05. **D** Venn diagram showing the overlapping relationships. The numbers indicate the gene counts among the DE-lncRNAs identified in 5 vs. 5 and 5 vs. 13 of MZ twins, and DE-lncRNAs identified from PyschENCODE brain RNA-seq data. **E** GO-BP enrichment analysis of mRNA targets of 4 DE-lncRNAs identified from our twin RNA-seq and independent PyschENCODE brain RNA-seq data

To identify DE-lncRNAs associated with bipolar disorder, we generated strand-specific RNA-seq datasets with a median of 100 million reads per sample from five pairs of BDC twins and four pairs of HCC twins (Additional file 1: Table S1), aligned the filtered reads to the UCSC *Homo sapiens* reference genome hg19 with TopHat2, and detected and characterized the expression patterns of the known annotated genes. Differential expression analysis of 6228 known lncRNAs with a 50th percentile count > 0 and 10th percentile count > 5 among nine MZ twin pairs identified 155 DE-lncRNAs that displayed significant expression differences (|Log_2_FC|> 0.585 and *P* < 0.05) in a pairwise analysis of 5 bipolar disorder vs. 5 healthy controls (5 BDC twins for 5 vs. 5; Fig. 1A, B). We then performed a case–control analysis of 5 bipolar disorders (from 5 BDC twins) vs. 13 healthy controls (by including 4 HCC twins, 5 vs. 13; Fig. 1C) and observed that 27 out of these 155 DE-lncRNAs remained significantly associated with bipolar disorder (|Log_2_FC|> 0.585 and *P* < 0.05; Fig. 1D and Additional file 1: Table S2). Among them, *AP1AR-DT*, *AC018647.1*, *ATP2C2-AS1*, and *AL596244.1* have been reported to be consistently differentially expressed in the postmortem brain tissue of bipolar disorder in the PsychENCODE brain RNA-seq dataset (*P* < 0.05; Fig. 1D and Table 1) [25]. We then performed expression correlation analysis of these four DE-lncRNAs with the mRNAs of the 18 individuals from the five BDC twins and four HCC twins and identified 6699 mRNAs correlated with at least one of these four lncRNAs (Pearson *p* < 0.05 and |*r*|> 0.5). Of these 6699 mRNAs, 2704 mRNAs were also reported to show bipolar disorder-associated expression changes (*P* < 0.05) in the PsychENCODE brain RNA-seq dataset [25]. Gene Ontology (GO) enrichment analysis of 2704 DE-mRNAs revealed that biological processes, such as neuron development, central nervous system development, and trans-synaptic signaling; cellular components, such as synapse, neuron projection, and postsynaptic density; and diseases, such as schizophrenia and bipolar disorder, were significantly enriched among mRNAs correlated with four DE-lncRNAs (Fig. 1E), indicating functional implications of these DE-lncRNAs in the pathogenesis of bipolar disorder.

**Table 1:** Bipolar disorder-associated DE-lncRNAs that were consistently altered in the blood of MZ twins and in post-mortem brains from the PsychENCODE RNA-seq dataset

Basic information		5 vs. 5 (5BDC)		5 vs. 13 (5BDC + 4HCC)		PsychENCODE (brain)	
**ID**	**Symbol**	**Log2(FC)**	***P* value**	**Log2(FC)**	***P* value**	**Log2(FC)**	***P* value**
ENSG00000260526	*AP1AR-DT*	0.9399	0.0113	0.8329	0.0494	0.0952	0.0019
ENSG00000261286	*ATP2C2-AS1*	1.2337	0.0086	1.3059	0.0493	0.1352	0.0261
ENSG00000261534	*AL596244.1*	− 1.1949	0.0297	− 1.5322	0.0191	− 0.0583	0.0399
ENSG00000227544	*AC018647.1*	− 1.2861	0.0385	− 1.7451	0.0233	− 0.1746	0.0051

### Gain of AP1AR-DT in mice leads depressive and anxiety-like states

Of the four identified DE-lncRNAs, *AP1AR-DT* (also called *AC109347.1, RP11-73K9.2*, *lnc-TIFA-1*, ENSG00000260526, or NONHSAG038649.2; chr4:112,229,561–112,231,596) was of particular interest for follow-up functional analyses, as it displays significant upregulation in the patients of BDC MZ twin in terms of gene (Log_2_(FC) = 0.9399 and *P* = 0.0113 for 5 vs. 5; and Log_2_(FC) = 0.8329 and *P* = 0.0494 for 5 vs.13; Table 1) and transcript levels based on FPKM (Log_2_(FC) = 1.2135, *P* = 0.017 for 5 vs. 5 and Log_2_(FC) = 0.7787, *P* = 0.021 for 5 vs.13) as well as in the post-mortem brain of bipolar disorder from an independent PsychENCODE brain RNA-seq dataset (Log_2_FC = 0.095 and FDR = 0.045) and maps to subthreshold GWAS regions (rs7667177 at chr4: 112,229,861, *P* = 5.2e-03) and adjacent to significant GWAS regions (rs13106460 at chr4: 118,385,980, *P* = 6.5e-06) associated with bipolar disorder in the PGC3 bipolar disorder GWAS [32]. The *AP1AR-DT* transcript exists as an isoform of 2036 nt with one exon in the human (Additional file 2: Fig. S1a) but has no corresponding sequences in the mouse. The in silico results obtained with the open reading frame (ORF) prediction tool ORFfinder to distinguish coding and non-coding transcripts consistently showed that *AP1AR-DT* has no potential protein coding ability (Additional file 2: Fig. S1b). We also observed that *AP1AR-DT* was moderately expressed in whole blood and brain tissues from GTEx RNA-seq datasets (Additional file 2: Fig. S1c). We then observed that *AP1AR-DT* was predominantly expressed in the nucleus (Additional file 2: Fig. S1d), as determined by RNA-seq in HeLa, MCF7, GM12878, or K562 cells, etc. from the lncATLAS dataset [33].

**Fig. 2:**
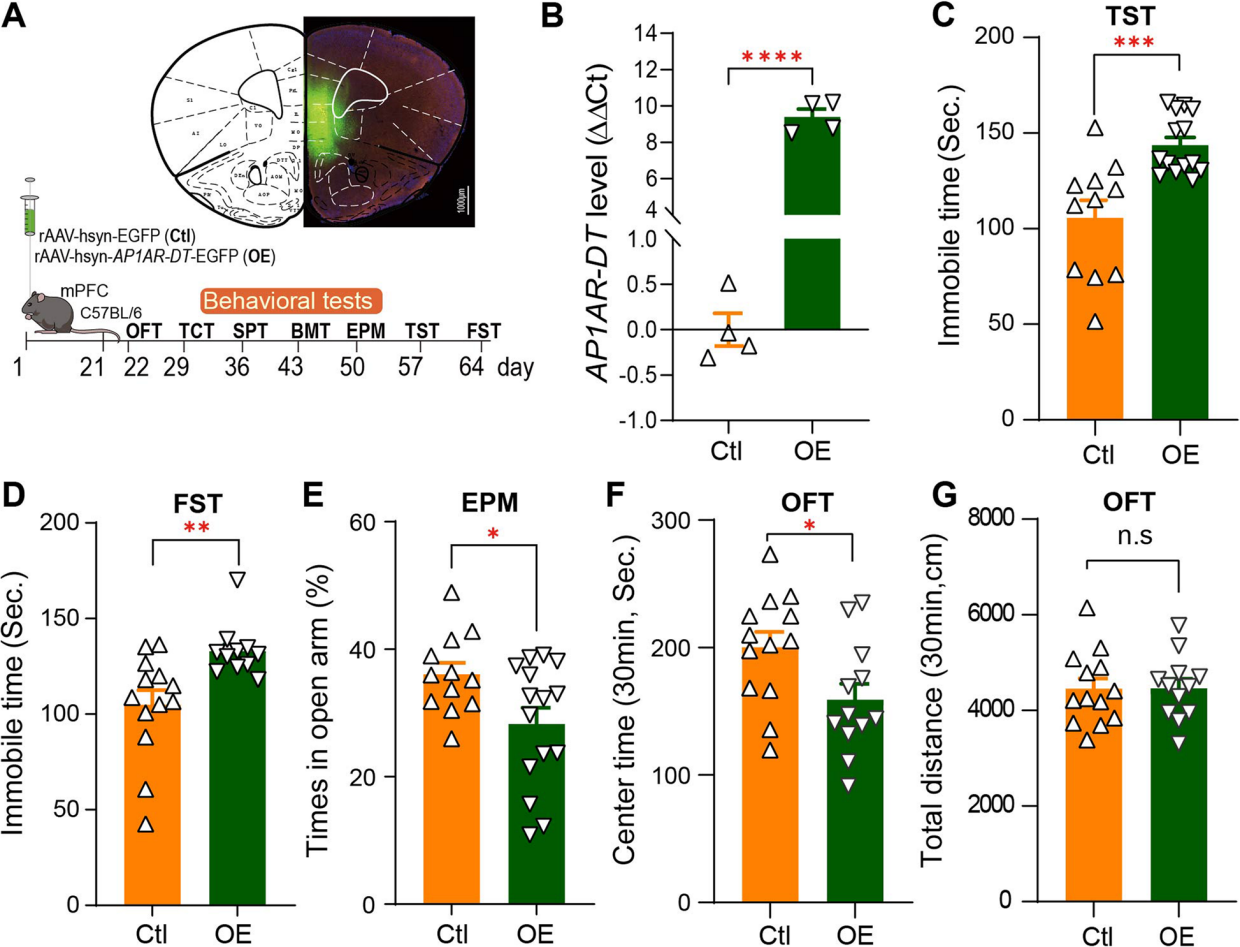
Overexpression of *AP1AR-DT* in mice leads to depressive and anxiety-like states. **A**, **B** Recombinant adeno-associated viruses (rAAVs) carrying lncRNA-*AP1AR-DT* (for *AP1AR-DT* overexpression, OE) or empty AAV vectors (as controls, Ctl) cloned into the AAV expression vector rAAV-hsyn-EGFP (green)-WPRE-hGHpA were injected into the medial prefrontal cortex (mPFC) regions of C57BL/6 wild-type mice (**A**), and their expression was examined by immunofluorescence analysis of brain slices (**A**) or by qRT-PCR shown as delta delta Ct values (**B**) The time line for each behavior test is also indicated. OFT, open field test; TCT, three-chamber test; SPT, sucrose preference test; BMT, Barnes maze test; EPM, elevated plus maze; TST, tail suspension test; FST, forced swimming test. **C**–**G ***AP1AR-DT* overexpressing mice showed depressive and anxiety-like behaviors. Depressive-like behaviors were measured by immobility time in the tail suspension test (**C**; TST, *n* = 11 Ctl and *n* = 14 OE mice) and the forced swim test (**D**; FST, *n* = 13 Ctl and *n* = 11 OE mice). Anxiety-like behavior was measured by time spent in the open arms of the elevated plus maze (**E**; EPM, *n* = 12 Ctl and *n* = 15 OE mice) and in the central area of the open field test (**F**, **G**; OFT, *n* = 13 Ctl and *n* = 12 OE mice). All data represent the means ± SEMs. A two-tailed *t*-test (*P < 0.05, **P < 0.01, ***P < 0.001. ns, nonsignificant) was used for comparisons between the two indicated groups

To investigate the function of bipolar disorder-related upregulated *AP1AR-DT*, we generated a recombinant AAV (rAAV) carrying *AP1AR-DT* (for *AP1AR-DT* overexpression, *AP1AR-DT*_OE_) driven under the human neuron specific synapsin I promoter and used an empty AAV vector as a control. Since disease-associated *AP1AR-DT* upregulation was observed in the cortex brain region from the PsychENCODE dataset and *AP1AR-DT* was also highly expressed in the human cortex brain based on the GETx dataset, we injected rAAV into the medial prefrontal cortex (mPFC) region of C57BL/6 male mice. Confocal images showed that the virus was accurately expressed in the mPFC neurons of the mice and an increased *AP1AR-DT* was successfully induced in the mice after 3 weeks of rAAV injection (Fig. 2A, B). We then performed a battery of behavior tests, including tail suspension test (TST), forced swim test (FST), elevated plus maze (EPM), open field test (OFT), three-chamber test, Barnes maze test, and sucrose preference test day 21 after rAAV injection (Fig. 2 A). Compared to the control mice, the *AP1AR-DT*_OE_ mice exhibited significantly increased immobility time in both the TST and FST (Fig. 2C, D), indicating a range of depression-like behaviors induced by *AP1AR-DT*_OE_. When tested for anxiety-like phenotype, *AP1AR-DT*_OE_ mice spent less time in the open arms and showed a preference for the near area than the control mice in the EPM test (*P* < 0.01, Fig. 2E) and spent less traveling time in the center of the OFT chamber with no difference in the total travel distance during the test session (30 min) than the control mice (Fig. 2F, G). However, *AP1AR-DT*_OE_ mice did not show significant differences in the three-chamber test, Barnes maze, and sucrose preference test as compared to the control mice (Additional file 2: Fig. S2). These behavioral data suggest that the gain of *AP1AR-DT* in mice leads to depressive and anxiety-like states.

### Gain of AP1AR-DT resulted in synaptic deficits

**Fig. 3:**
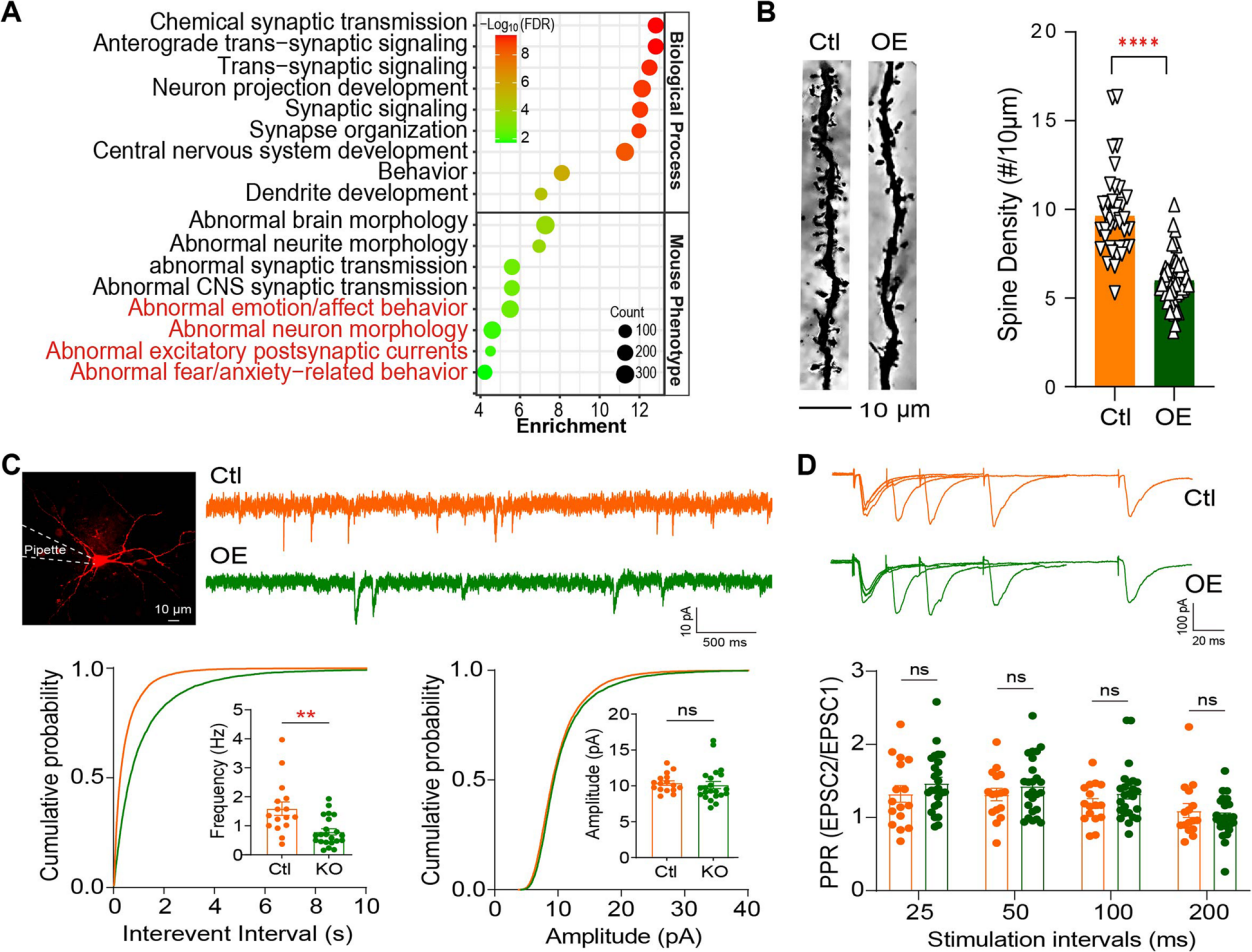
Gain of *AP1AR-DT* resulted in synaptic deficits. **A** Functional enrichment analysis of 2782 protein-coding DEGs (*P* < 0.05) induced by *AP1AR-DT*_*OE*_ in mice from RNA-seq. **B** Spine densities were significantly decreased in *AP1AR-DT*_*OE*_ mice compared to control mice. *n* = 46 neurons from five *AP1AR-DT*_*OE*_ mice and 39 neurons from five control mice. **C** Representative recording traces (top) and cumulative probability plots of spontaneous excitatory postsynaptic currents (sEPSCs; bottom) showing that the frequencies (*P* = 0.0015) but not amplitudes were decreased in *AP1AR-DT*_*OE*_ mPFC pyramidal neurons. *n* = 16 cells from four control mice and *n* = 22 cells from three *AP1AR-DT*_*OE*_ mice. **D** Representative traces of pair-pulse stimulation responses (top) and pair-pulse ratios (PPRs) plotted against interstimulus intervals (bottom) show that PPRs remained unchanged. *n* = 22 cells from four *AP1AR-DT*_*OE*_ mice and *n* = 16 cells from three control mice. All data represent the means ± SEMs. A two-tailed t-test (**P < 0.01, ****P < 0.0001, ns, nonsignificant) was used for comparisons between the two indicated groups

To elucidate the underlying molecular mechanisms of *AP1AR-DT*_*OE*_-induced anxiety- and depression-like states in mice, we performed RNA-seq using RNA extracted from mPFCs of *AP1AR-DT*_*OE*_ mice or control mice to identify DEGs. Transcriptome analyses identified 2415 DEGs (|Log2FC|> 0.32, *P* < 0.05), including 1110 upregulated and 1305 downregulated DEGs in *AP1AR-DT*_*OE*_ mice compared with control mice. Gene Ontology enrichment analysis of those *AP1AR-DT*_*OE*_-induced mouse DEGs were involved in biological processes such as chemical synaptic transmission, anterograde trans-synaptic signaling, neuron projection development, synaptic signaling, and synapse organization and mouse phenotypes such as abnormal brain morphology, abnormal neurite morphology, abnormal emotion/affect behavior, and abnormal fear/anxiety-related behavior and abnormal locomotor activation (Fig. 3A), most of which have been reported to be associated with bipolar disorder, further providing functional implication of *AP1AR-DT* in the pathogenesis.

Considering that *AP1AR-DT*_*OE*_-induced mouse DEGs are associated with neuronal development or chemical synaptic transmission in functional enrichment analysis, we thus performed Golgi staining using mouse mPFC tissue at postnatal day 90 to determine whether *AP1AR-DT*_*OE*_ affects dendritic structure in vivo. Compared to the neurons in the mPFC region of control mice, the neurons from the *AP1AR-DT*_*OE*_ mice exhibited a reduction in the total spine density (stubby and mushroom-shaped spine types; Fig. 3B). These results suggest that *AP1AR-DT* plays an essential role in regulating synaptic structure or function. Next, we determined whether *AP1AR-DT* alters synaptic function by performing a whole-cell recording electrophysiological assay on acute brain slices. We observed that the spontaneous excitatory postsynaptic current (sEPSC) frequency, but not amplitude, of *AP1AR-DT*_*OE*_ pyramidal neurons was significantly decreased (Fig. 3C). The decreased sEPSC frequency is likely due to the reduction in the synapse number rather than presynaptic neurotransmitter release, because the pair-pulse ratios (PPRs) at 25-, 50-, and 100-ms intervals remained unchanged for *AP1AR-DT*_*OE*_ neurons compared to control neurons (Fig. 3D). Collectively, these results indicate that *AP1AR-DT* plays a vital role in maintaining proper synaptic function.

### Repression of NEGR1 by AP1AR-DT through competing binding to the NEGR1 promoter with the transcriptional activator NRF1

**Fig. 4:**
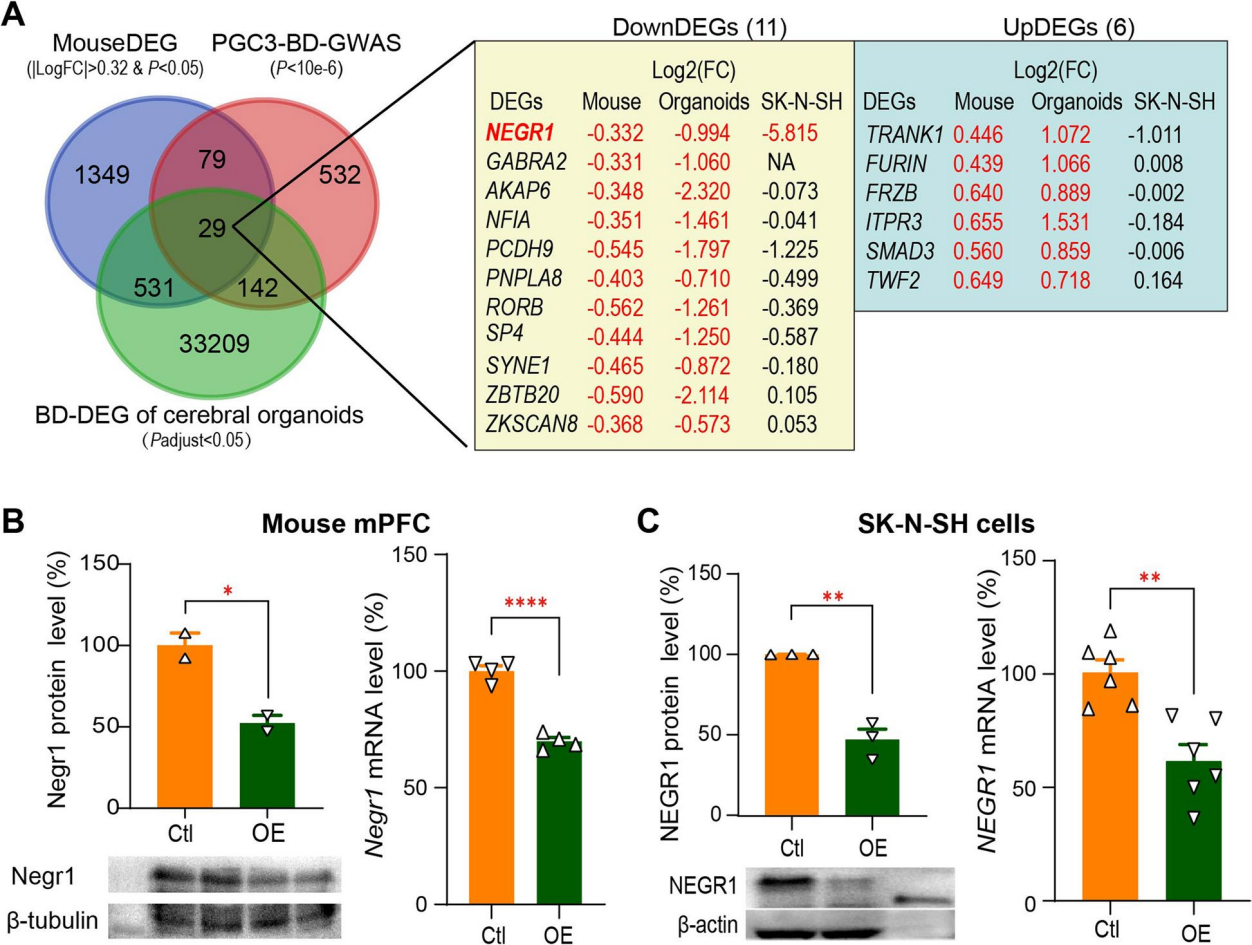
Targets of *AP1AR-DT* identification in vivo*. ***A** Eleven downregulated (DownDEGs) and six upregulated (UpDEGs) potential targets of *AP1AR-DT* were identified by overlapping the *AP1AR-DT*_*OE*_-induced mouse DEGs, bipolar disorder-associated DEGs from human iPSC-induced cerebral organoids, and bipolar disorder-associated genes identified in the PGC3-GWAS. Log2(FC) are shown for each gene from *AP1AR-DT*_*OE*_ mouse, bipolar disorder-associated DEGs from human iPSC-induced cerebral organoids and *AP1AR-DT*_*OE*_ SK-N-SH cells, with red font denoting DEG with *P* < 0.05. **B**, **C** The protein (left) and RNA (right) levels of *Negr1* in mPFC of *AP1AR-DT*_*OE*_ mouse (**B**) and in *AP1AR-DT*_*OE*_ SK-N-SH cell lines (**C**). All data represent the means ± SEMs. A two-tailed t-test (*P < 0.05, **P < 0.01, ****P < 0.0001) was used for comparisons between the two indicated groups

We then examined whether *AP1AR-DT*_*OE*_-induced mouse DEGs are orthologous to the reported bipolar disorder-associated genes. Notably, we observed that the *AP1AR-DT*_*OE*_-induced mouse coding DEGs were significantly enriched in the PGC3 bipolar disorder GWAS genes [32] (29 + 79 overlapping genes; enrichment OR = 1.50 and *P* = 2.8e–4) and in the bipolar disorder-associated DEGs in the cerebral organoid RNA-seq dataset (29 + 531 overlapping genes; enrichment OR = 1.75 and* P* = 4.9e–24; Fig. 4A) [34]. Of these 29 overlapping genes, 11 genes are consistently downregulated and 6 genes are consistently upregulated in both *AP1AR-DT*_*OE*_ mice and bipolar disorder cerebral organoids. In particular, the *Negr1* and *Trank1* are involved in neuronal development and differentiation and synaptic plasticity and have been reported to be associated with bipolar disorder or major depressive disorder [27, 35–38], which may partially explain the depressive and anxiety-like behaviors or synaptic deficits in *AP1AR-DT*_*OE*_ mice. We then overexpressed *AP1AR-DT* via a recombinant lentiviral vector in human SK-N-SH neuroblastoma cells and performed RNA-seq analysis of *AP1AR-DT* overexpressed SK-N-SH cells (Log_2_FC = 6.630 and *P* = 4.11E–25) to verify the *AP1AR-DT*-induced DEGs. We observed that out of the above 17 genes, only *NEGR1* displays a significant reduction in the *AP1AR-DT*_*OE*_ SK-N-SH cell lines (Log_2_FC = -5.82 and *P* = 0.026; Fig. 4A), consistent with the observations from *AP1AR-DT*_*OE*_ mouse (Log_2_FC = -0.332 and *P* = 0.022) and the bipolar disorder cerebral organoid RNA-seq dataset (Log_2_FC = – 0.994 and *P* = 0.003). *NEGR1* also shows bipolar disorder-associated reductions in the PsychENCODE brain RNA-seq dataset at the transcript level (ENST00000434200, Log_2_FC = – 0.822 and *P* = 0.009). Furthermore, *AP1AR-DT*_*OE*_-induced downregulation of NEGR1 was also validated at the protein and mRNA levels in *AP1AR-DT*_*OE*_ mouse mPFC brain (Fig. 4B) and SK-N-SH cells (Fig. 4C) using Western blotting and qPCR assays. *NEGR1* (ENSG00000172260; chr1:71,395,943–72,282,539; GRCh38) encodes neuronal growth regulator 1, a synaptic adhesion molecule that has been implicated in human major depressive disorder in several studies [27, 37, 39, 40]. Negr1 deficiency also leads to an anxiety- and depression-like state in mice and is accompanied by reduced adult neurogenesis and excitatory synaptic function [27, 38], which is consistent with our observation in mice with gain of *AP1AR-DT*.

**Fig. 5:**
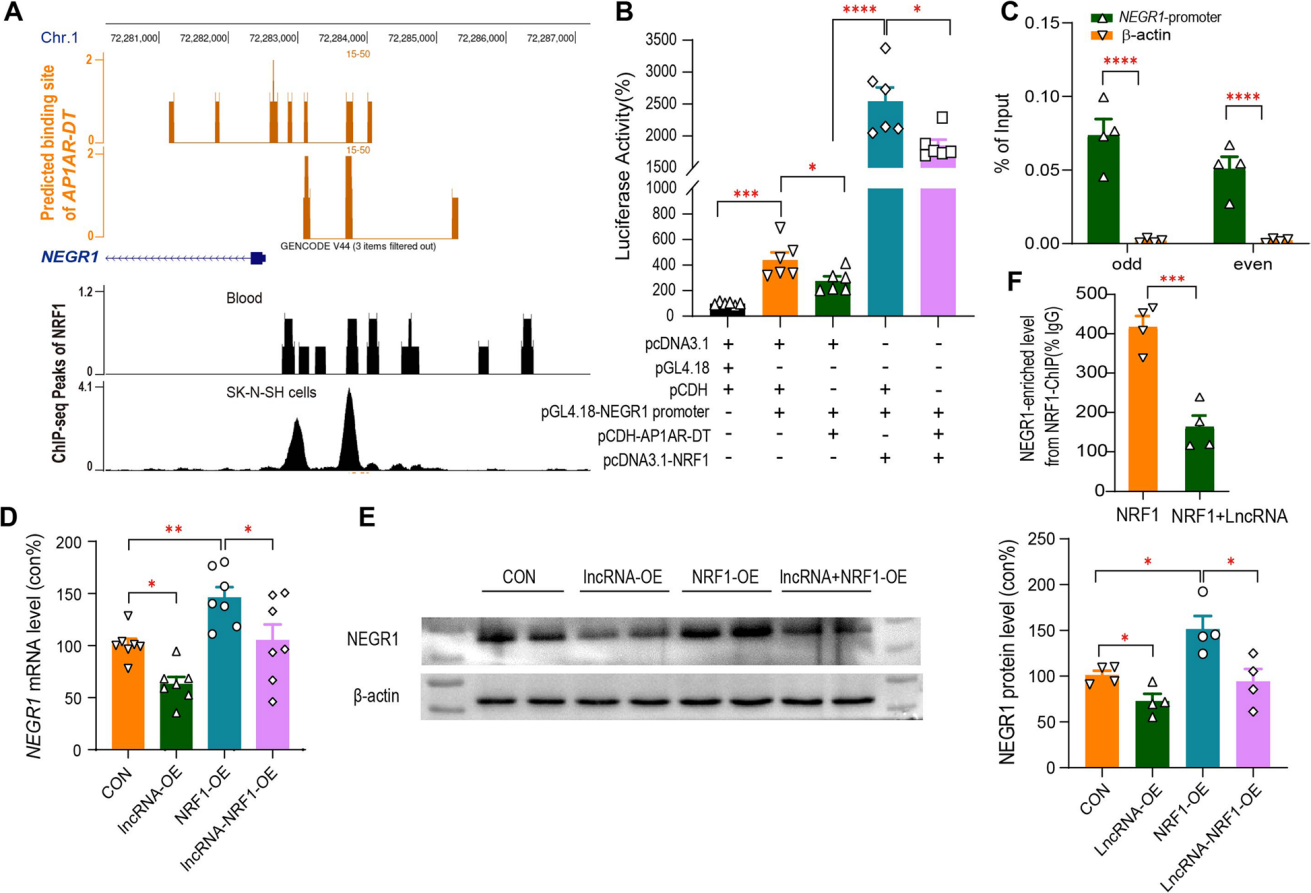
Repression of NEGR1 by *AP1AR-DT* through competing binding to the *NEGR1* promoter with NRF1. **A** The putative binding regions of *AP1AR-DT* in the *NEGR1* upstream region were predicted using LongTarget (top, orange), and the binding peaks of NRF1 in the *NEGR1* upstream region were obtained from anti-NRF1 ChIP-seq analysis of blood and SK-N-SH cell samples (bottom, black). **B** The effects of *AP1AR-DT* and NRF1 on the *NEGR1* promoter activity were examined by co-transfection of a dual-luciferase reporter containing the *NEGR1* promoter region (from − 1149 to − 1246) in the pGL4.18 vector with the pCDH vector containing *AP1AR-DT* or the pcDNA3.1 vector containing NRF1 coding region in HEK293T cells. **C** ChIRP assay of the interaction of *NEGR1* with *AP1AR-DT* in the *AP1AR-DT*_*OE*_ HEK293T cells. The retrieved *NEGR1* DNA from the *AP1AR-DT* ChIRP analysis was quantified by qPCR and is expressed as the percentage of input in columns. The β-actin (orange) served as a negative control. Green: odd-numbered probes (#1, 3, 5); green: even-numbered probes (#2, 4, 6). **D**, **E** The effects of *AP1AR-DT* and NRF1 on the endogenous *NEGR1* RNA (**D**) and protein (**E**) levels in the SK-N-SH cell lines transfected with the pCDH-*AP1AR-DT* (lncRNA-OE) or/and pcDNA3.1-NRF1 (NRF1-OE) vectors. **F** ChIP-qPCR analysis of NRF1 enrichment at the *NEGR1* promoter in the SK-N-SH cells cotransfected with pcDNA3.1-NRF1 without (orange) or with (green) pCDH-*AP1AR-DT*. All data represent the means ± SEMs. A two-tailed *t*-test was used for comparisons between the two indicated groups (*P < 0.05, **P < 0.01, ***P < 0.001, and ****P < 0.0001)

LncRNAs can inhibit gene transcription by binding to DNA, and forming RNA:DNA triplexes and preventing the binding of transcription factors (TFs) to the promoter. We used LongTarget [41] to predict several potential binding sites of *AP1AR-DT* in the promoter regions of *NEGR1* (Fig. 5A). We then validated the downregulation of *NEGR1* promoter activity by *AP1AR-DT*_OE_ in HEK293T cells cotransfected with pCDH-*AP1AR-DT* and a pGL4.18 luciferase reporter vector driven by the *NEGR1* promoter (Fig. 5B). Next, we examined the binding activities of *AP1AR-DT* to the *NEGR1* promoter region using chromatin isolation by RNA purification (ChIRP) assay and observed that *AP1AR-DT* can bind to the upstream of the transcription start site spanning from 1149 ~ 1246 bp promoter region of *NEGR1*, as determined by qPCR analysis of the retrieved DNA from *AP1AR-DT*-ChIRP (Fig. 5C). We further used ENCODE TF chromatin immunoprecipitation (ChIP)-seq data to screen for potential TFs that bind to the *NEGR1* promoter region and observed that nuclear respiratory factor 1 (NRF1, ENSG00000106459; chr7: 129,611,720–129,757,082; GRCh38) exhibited significant binding peaks within this region overlapping with the *AP1AR-DT* binding site based on ChIP-seq of the SK-N-SH cell line (Fig. 5A). In addition, NRF1-binding motifs (CAGCGCGCCTGCGTG, GCGCGTGCACA) were also predicted in the *NEGR1* promoter region overlapping with the *AP1AR-DT* binding region by JASPAR online software [42] (Additional file 2: Fig. S3). NRF1 functions as a transcriptional activator and has been reported to be involved in the regulation of neurite outgrowth [43, 44]. We then verified that NRF1 could enhance the *NEGR1* promoter activity, which was suppressed by the *AP1AR-DT*_*OE*_, in HEK293T cells cotransfected with pCDH-*AP1AR-DT* or pcDNA3.1-NRF1 and a pGL4.18 luciferase reporter vector driven by the *NEGR1* promoter (Fig. 5B). We also verified that NRF1 overexpression significantly increased endogenous *NEGR1* mRNA (Fig. 5D) and protein (Fig. 5E) levels, which was inhibited by the *AP1AR-DT*_*OE*_ in SK-N-SH cells. Finally, we verified that NRF1 exhibited binding activity to the *NEGR1* promoter region, which was significantly reduced by the *AP1AR-DT*_*OE*_ in SK-N-SH cells, as determined by qPCR analysis of DNA retrieved from the NRF1-ChIP assay (Fig. 5F). Collectively, these results suggest that *AP1AR-DT* reduces NEGR1 expression by competing for the transcriptional activator NRF1 in the overlapping binding site of the *NEGR1* promoter region.

### Rescue of Negr1 expression in mice ameliorated AP1AR-DTOE induced behavioral and synaptic deficits

**Fig. 6:**
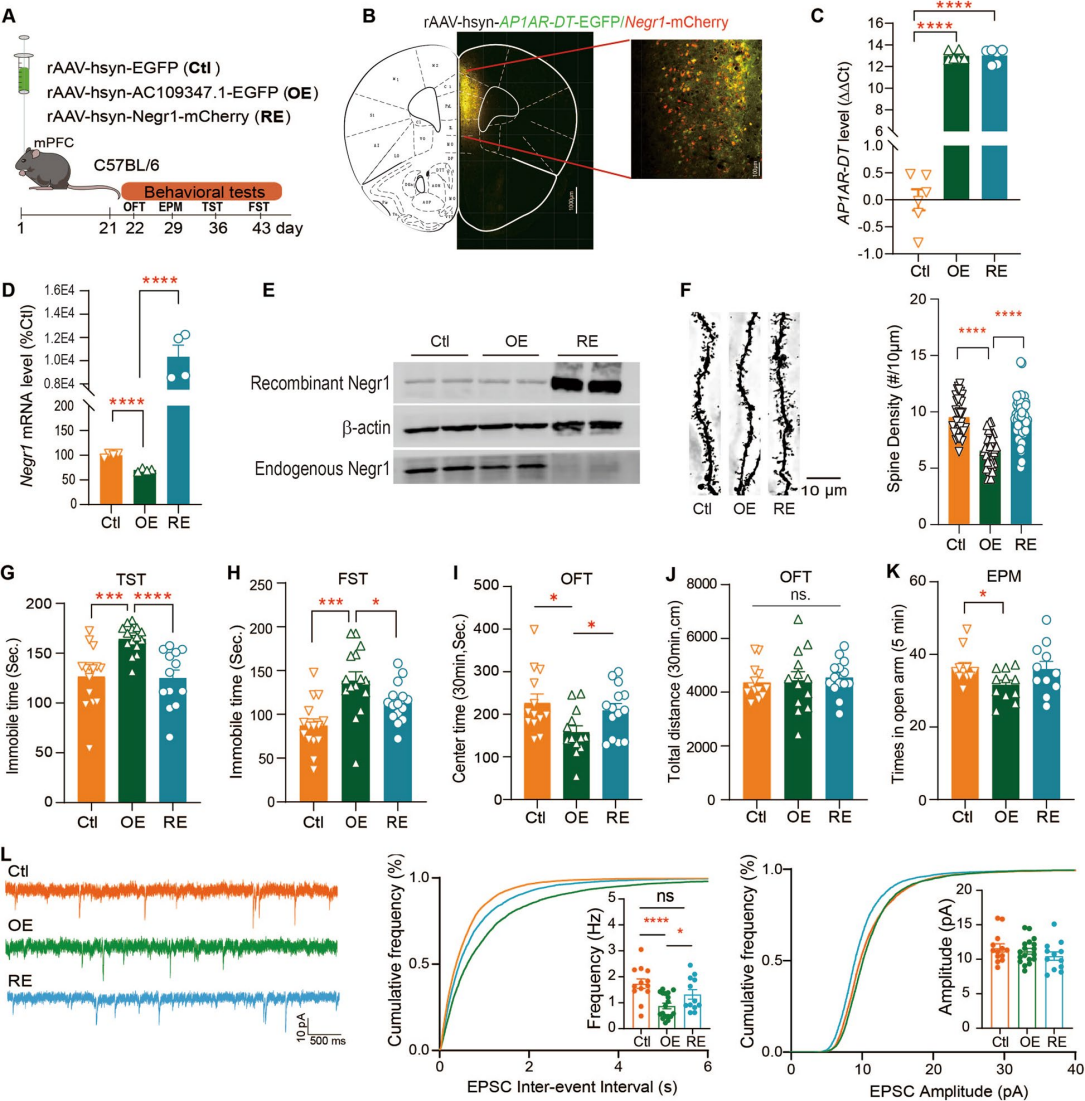
Rescue of *Negr1* expression ameliorates behavioral and synaptic deficits induced by the gain of *AP1AR-DT* in mice. **A**–**E** rAAVs carrying lncRNA-*AP1AR-DT* (OE), *Negr1*(for rescue of *Negr1* expression, *RE*), or empty rAAV vectors (Ctl) were cloned into the rAAV expression vector and injected into the mPFC regions of C57BL/6 wild-type mice (**A**), and their expression levels were examined by immunofluorescence analysis of brain slices (**B**) or by qRT-PCR analysis of *AP1AR-DT* (**C**) and the *Negr1* mRNA (**D**) levels and Western blotting analysis of Negr1 protein levels using an anti-mCherry antibody for recombinant Negr1 and an anti-Negr1 antibody for endogenous Negr1(**E**). The time line for each behavior test is also indicated. **F** The reduced spine densities in *AP1AR-DT OE* mice (OE) neurons were rescued in Negr1 rescued mice (RE). *n* = 35 neurons from Ctl mice, *n* = 36 neurons from OE mice, and *n* = 48 neurons from RE mice. **G**–**K** Rescue of *Negr1* expression in *AP1AR-DT OE* mice restored the immobile times of TST (*n* = 15 Ctl mice, *n* = 15 OE mice, and *n* = 13 RE mice, **G**) and FST (*n* = 15 Ctl mice, *n* = 15 OE mice, and *n* = 15 RE mice, **H**), time spent in the central area of the OFT (*n* = 13 Ctl mice, *n* = 13 OE mice, and *n* = 14 RE mice, **I**–**J**), and time spent in the open arms of the EPM (*n* = 11 Ctl mice, *n* = 11 OE mice, and *n* = 11 RE mice, **K**). **L** Rescue of *Negr1* expression in *AP1AR-DT OE* mice ameliorates the impaired sEPSCs induced by *AP1AR-DT*_*OE*_*.* Representative recording traces and cumulative probability plots of sEPSCs showing that the decreased sEPSC frequency in *AP1AR-DT*_*OE*_ mice (OE) was increased when the Negr1 expression was restored (RE) in mPFC neurons. *n* = 13 cells from four Ctl mice, *n* = 18 cells from three OE mice, and *n* = 12 cells from three RE mice. All data represent the means ± SEMs. A two-tailed *t*-test was used for comparisons between the two indicated groups (*P < 0.05, **P < 0.01, ***P < 0.001, and ****P < 0.0001. ns, nonsignificant)

Since significantly reduced Negr1 RNA and protein levels were observed in the *AP1AR-DT*_*OE*_ mice, we next investigated whether rescue of Negr1 expression would reduce the behavioral and synaptic deficits associated with the gain of *AP1AR-DT* in mice. To do this end, we injected the rAAV carrying *Negr1* under the human synapsin I promoter into the mPFC regions of C57BL/6 *AP1AR-DT*_*OE*_ mice (*Negr1*-RE mice, RE), allowing restoration of *Negr1* expression in neurons (Fig. 6A). We first verified that the reduced Negr1 expression caused by *AP1AR-DT*_*OE*_ was restored in *Negr1*-RE mice (Fig. 6B–E). We then observed that the reduced spine density in *AP1AR-DT*_*OE*_ mice was ameliorated when Negr1 expression was rescued in mPFC neurons of *Negr1-*RE mice (Fig. 6F). We next examined whether the rescue of *Negr1* ameliorated the abnormal behaviors observed in *AP1AR-DT*_*OE*_ mice. We observed that *Negr1-*RE ameliorated depression-like behaviors in *AP1AR-DT*_*OE*_ mice, as evidenced by reduced immobility time in the TST and FST as compared to the *AP1AR-DT*_*OE*_ mice (Fig. 6G, H). In addition, *Negr1-*RE also improved anxiety-like behaviors, showing increased travel time in the central of the OFT chamber (Fig. 6 I)  but no difference in the total travel distance during the test session (30 min; Fig. 6J) and in times in the open arm of the EPM test (Fig. 6K), compared to the *AP1AR-DT*_*OE*_ mice. Finally, we investigated whether the synaptic function deficits in *AP1AR-DT*_*OE*_ mice could be ameliorated in *Negr1-*RE mice. We observed that *Negr1-*RE mice had similar frequencies of spontaneous excitatory postsynaptic currents (sEPSCs) to the control mice, both of which had higher frequencies of sEPSCs than the *AP1AR-DT*_*OE*_ mice, and similar sEPSC amplitudes (Fig. 6L) and PPRs (Additional file 2: Fig. S4) to the control and *AP1AR-DT*_*OE*_ mice. Taken together, these results suggest that depressive- and anxiety-like behaviors in *AP1AR-DT*_*OE*_ mice can be attenuated by normalizing the excitatory synaptic transmission of mPFC neurons through restoring of the Negr1 activity.

## Discussion

Bipolar disorder is a complex polygenic disorder that is characterized by a combination of periodic episodes of mania and depression. LncRNAs exert their regulatory function by interacting with DNA, RNA, or proteins of numerous targets via a structural domain formed by complementary base pairing and transcript folding. Altered lncRNA profiles have been implicated in bipolar disorder [25, 32, 34]. However, the functional contribution of molecular networks and interactions among many targets of implicated lncRNAs to the pathogenesis of bipolar disorder remains largely unknown. In this study, we employed a twin design to identify DE-lncRNAs associated with disease susceptibility in MZ twins. Because phenotypically discordant MZ twins share a common genetic background, profiling of MZ twins to control for similar genetic backgrounds increases the power of this study of phenotypic variation through changes in epigenetic profiles. We then employed several robust methodologies to interpret the findings in patients with bipolar disorder in an animal model of the disease. This revealed the epigenetic mechanism by which the gain of bipolar disorder-associated upregulated *AP1AR-DT* in mice induces depressive and anxiety-like behaviors by reducing Negr1-mediated excitatory synaptic transmission, providing an etiological implication for bipolar disorder.

In this study, RNA-seq analysis of peripheral blood from five pairs of BDC twins revealed 155 bipolar disorder-associated DE-lncRNAs. Furthermore, 27 lncRNAs remained consistently altered in bipolar disorder after four HCC twin pairs were included to control for the non-psychiatric trait differences between MZ twins. This approach was also used in our recent studies [26, 45]. Notably, results from an independent post-mortem brain RNA-seq dataset from PsychENCODE [25] demonstrated that four out of 27 lncRNAs associated with bipolar disorder were consistently altered in bipolar disorder. This finding supports that a study of epigenetic profiles in a small number of MZ twins can successfully identify epigenetic variations associated with complex traits. Among the four bipolar disorder-associated DE-lncRNAs identified in MZ twins, *AP1AR-DT* exhibits a consistent bipolar disorder-associated increase in both blood and in postmortem brain tissue (FDR = 0.045) in a previous publication [25]. Furthermore, *AP1AR-DT* is located within the subthreshold GWAS regions and in close proximity to significant GWAS regions associated with bipolar disorder in the PGC3 bipolar disorder GWAS [32]. Furthermore, our findings demonstrated that overexpression of *AP1AR-DT* in the mouse mPFC induces depression and anxiety-like behaviors. This is further supported by the functional annotation of *AP1AR-DT* OE-induced mouse DEGs in abnormal affect and anxiety-related behaviors as well as chemical synaptic transmission and neuronal projection development. Subsequently, we demonstrated that *AP1AR-DT*_*OE*_ mice exhibited a reduction in both the total spine density of mPFC neurons and the sEPSC frequency of mPFC pyramidal neurons. This suggests that *AP1AR-DT* plays an important role in maintaining proper synaptic function, which is involved in the pathogenesis of bipolar disorder.

We have delineated the epigenetic mechanism by which the gain of *AP1AR-DT* in mice induces depression and anxiety-like behaviors by reducing Negr1-mediated excitatory synaptic transmission. Changes in dendritic structure and synaptic organization are associated with thousands of proteins and are ultimately responsible for learning, memory, cognition, and other brain functions that are also implicated in bipolar disorder. Therefore, it is critical to identify the gene networks that modulate dendritic structure, synaptic transmission, and neuronal development. Our findings indicated that *AP1AR-DT*_*OE*_-induced mouse DEGs are involved in a multiple of pathways that regulate synaptic plasticity and excitability. Enrichment analysis of *AP1AR-DT*_*OE*_-induced DEGs in both mice and SK-N-SH cells and bipolar disorder-associated genes revealed that neuronal growth regulator 1 (Negr1), a synaptic adhesion molecule, is a potential target of *AP1AR-DT*. Furthermore, its downregulation by *AP1AR-DT*_*OE*_ was verified in mouse brain and SK-N-SH cells. *NEGR1* plays a role in axon extension, synaptic plasticity, and synapse formation, which are key processes for neuronal function [46, 47]. *NEGR1* has been reported to be associated with major depressive disorder in several GWAS [37, 39, 40, 48] and to be downregulated in the CA1 and dentate gyrus (DG) regions in major depressive disorder [40]. More recently, Negr1-deficient mice exhibited both anxiety- and depressive-behaviors as well as a reduction in miniature EPSC frequency in DG granule cells [38, 47]. Notably, these phenotypes were all recapitulated in the *AP1AR-DT*_*OE*_ mice in our study. Moreover, the overexpression of recombinant Negr1 in *AP1AR-DT*_*OE*_ mice resulted in the amelioration of anxiety- and depressive-like behaviors as well as the normalization of the reduced excitatory synaptic transmission induced by the gain of *AP1AR-DT*. Furthermore, we demonstrated that the downregulation of *NEGR1* by *AP1AR-DT* prevents the binding of the transcriptional activator NRF1 to the *NEGR1* promoter. Nuclear respiratory factor 1 (NRF1), a redox-sensitive positive regulator of transcription, has been reported to functionally regulate critical NMDA receptor subunit genes in response to changes in neuronal activity [49]. In addition, transcriptomic analysis of *AP1AR-DT*_*OE*_ mouse mPFC revealed that 902 out of 2415 mouse DEGs displayed bipolar disorder-associated expression changes (*P* < 0.05) in the PsychENCODE brain RNA-seq dataset [25] or in the cerebral organoid RNA-seq dataset [34], including *TRANK1, FURIN*, *GABRA2*, and *SYNE1,* which are involved in modulating chemical synaptic transmission, trans-synaptic signaling, and synaptic signaling pathways. Collectively, these findings provide insight into the regulatory roles of *AP1AR-DT* in gene expression associated with synaptic dysfunction. This knowledge may inform the development of promising interventions for bipolar disorder.

Although overexpression of *AP1AR-DT* in mouse mPFC neurons using the AAV system has been demonstrated to induce anxiety and depressive-like phenotypes, a conditional genetic modulation model that allows for the conditionally overexpression of *AP1AR-DT* in specific neuron types may be a more optimal choice for future studies. Given that the *AP1AR-DT* upregulation associated with bipolar disorder was identified in our study and also verified in postmortem brain tissue in another independent study, further studies are required to establish the links between dysregulated *AP1AR-DT* in the peripheral system and modulation of excitatory synaptic transmission in a rodent mouse model. It is notable that *TRANK1*, one of the most robust bipolar disorder risk genes [4, 35, 50, 51], showed significant upregulation in *AP1AR-DT*_*OE*_ mouse brain and in bipolar disorder cerebral organoids. Recent reports have implicated microbiota-gut-brain regulation in the neuropathogenesis of bipolar depression by modulating TRANK1 expression [36], suggesting that Trank1 may be another potential target for future study to examine the functional links between the dysregulated *AP1AR-DT* identified in the peripheral system and the neuropathogenesis of bipolar disorder. Further studies are required to confirm the role of *AP1AR-DT* in the pathophysiology of bipolar disorder, particularly in a mania-like phenotype, as this is another prevalent symptom of the disorder. It would also be beneficial to validate the functions of other DE-lncRNAs identified in this study.

## Conclusions

We employed BDC MZ twins to screen for DE-lncRNAs associated with susceptibility to bipolar disorder in MZ twins. Our findings illustrate an epigenetic mechanism by which the gain of bipolar disorder-associated upregulated *AP1AR-DT* in mice induces depressive and anxiety-like behaviors by reducing Negr1-mediated excitatory synaptic transmission. The epigenetic and pathophysiological mechanism linking *AP1AR-DT* to the modulation of excitatory synaptic function provides etiological implications for bipolar disorder.

## Supplementary Information

Additional file 1: Tables S1-S3. Table S1- [Summary of RNA-seq data for each twin pair]. Table S2- [27 DE-lncRNAs identified from twins]. Table S3- [Primers and probes employed in this study].

Additional file 2: Figs. S1-S4. Fig S1- [The information of *AP1AR-DT*]. Fig S2- [Behavioral results of *AP1AR-DT* overexpression in the three-chamber test, Barnes maze test and sucrose preference test]. Fig S3- [The location of binding sequence of *AP1AR-DT* to *NEGR1* promoter region and every primer sequence on promoter region]. Fig S4- [Representative traces of pair-pulse stimulation responses and pair-pulse ratios plotted against interstimulus intervals].

Additional file 3. Original images for Figs. 4B, 4C, 5E and 6E.

## Data Availability

All the data generated in this study were shown in the main text and supplementary material. RNA sequencing data from AP1AR-DT_OE_ SK-N-SH cells (GSE272752) and AP1AR-DT_OE_ mice mPFC (GSE272753) have been deposited to the NCBI GEO site. Additional information is also available upon reasonable request to the corresponding authors.

## Abbreviations

MZ: Monozygotic
mPFC: Medial prefrontal cortex
GWAS: Genome-wide association studies
lncRNA: Long non-coding RNA
TDP-43: TAR DNA-binding protein-43
DLPFC: Dorsolateral prefrontal cortex
BDC: Bipolar disorder discordant
HCC: Healthy concordant control
FPKM: Per kilobase of transcript per million mapped reads
FC: Fold change
DE: Differentially expressed
DEGs: Differentially expressed genes
GO: Gene Ontology
HEK: Human embryonic kidney
pCDH: TK-pCDH-copGRP-T2A-Puro
rAAV: Recombinant adeno-associated virus
OFT: Open-field test
TCT: Three-chamber test
EPM: Elevated plus maze
TST: Tail suspension test
FST: Forced swim test
ChIRP: Chromatin isolation by RNA purification
ChIP: Chromatin immunoprecipitation
sEPSC: Spontaneous excitatory postsynaptic currents
NEGR1: Neuronal growth regulator 1
