# Supplementary material for: Gain of bipolar disorder-related lncRNA AP1AR-DT in mice induces depressive and anxiety-like behaviors by reducing Negr1-mediated excitatory synaptic transmission
Source: BMC Med. 2024 Nov 18;22:543. doi: 10.1186/s12916-024-03725-0 (PMC11575081; doi:10.1186/s12916-024-03725-0)
Supplement: Supplementary file 1 — Additional file 1: Tables S1-S3. Table S1- [Summary of RNA-seq data for each twin pair]. Table S2- [27 DE-lncRNAs identified from twins]. Table S3- [Primers and probes employed in this study]. [file 12916_2024_3725_MOESM1_ESM.docx]

| **Table S1 Summary of RNA-seq data for each twin pair** | | | | | |  |  |  |  |
| --- | --- | --- | --- | --- | --- | --- | --- | --- | --- |
|  | **Sample** | **Diagnosis** | **Gender** | **Old (years)** | **Total number of reads** | **Mapped reads** | **Unique mapped reads** | **Percentage of mapped reads in total reads** | **Percentage of unique mapped reads in mapped reads** |
| **PDC twins** | **BDC1a** | **Biplolar disorders (BPD)** | Male | 44 | 99362902 | 94124718 | 86365232 | 94.73% | 91.76% |
|  | **BDC1u** | **Healthy** |  |  | 102308798 | 95012788 | 86809882 | 92.87% | 91.37% |
|  | **BDC2a** | **Biplolar disorders (BPD)** | Male | 17 | 190029316 | 169683648 | 151597650 | 89.29% | 89.34% |
|  | **BDC2u** | **Healthy** |  |  | 140166150 | 124640308 | 111150112 | 88.92% | 89.18% |
|  | **BDC3a** | **Biplolar disorders (BPD)** | Male | 18 | 103077580 | 98431034 | 80515990 | 95.49% | 81.80% |
|  | **BDC3u** | **Healthy** |  |  | 104432628 | 99891774 | 79301332 | 95.65% | 79.39% |
|  | **BDC4a** | **Biplolar disorders (BPD)** | Male | 22 | 102561710 | 96997274 | 81560042 | 94.57% | 84.08% |
|  | **BDC4u** | **Healthy** |  |  | 105732952 | 99783224 | 87625596 | 94.37% | 87.82% |
|  | **BDC5a** | **Biplolar disorders (BPD)** | Female | 18 | 140995872 | 133143124 | 104997366 | 94.43% | 78.86% |
|  | **BDC5u** | **Healthy** |  |  | 98389832 | 92058940 | 73807250 | 93.57% | 80.17% |
| **HCC twins** | **HCC1a** | **Healthy control (HCC)** | Female | 21 | 182054370 | 150172400 | 138238948 | 82.49% | 75.93% |
|  | **HCC1b** | **Healthy control (HCC)** |  |  | 165497252 | 136238930 | 124205616 | 82.32% | 75.05% |
|  | **HCC2a** | **Healthy control (HCC)** | Male | 26 | 132186612 | 108824428 | 101783752 | 82.33% | 77.00% |
|  | **HCC2b** | **Healthy control (HCC)** |  |  | 158811382 | 126231062 | 117150848 | 79.48% | 73.77% |
|  | **HCC3a** | **Healthy control (HCC)** | Male | 23 | 137436792 | 115508998 | 108925640 | 84.05% | 79.26% |
|  | **HCC3b** | **Healthy control (HCC)** |  |  | 133513884 | 107835694 | 102062194 | 80.77% | 76.44% |
|  | **HCC4a** | **Healthy control (HCC)** | Female | 25 | 96093112 | 80982354 | 75490862 | 84.27% | 78.56% |
|  | **HCC4b** | **Healthy control (HCC)** |  |  | 97347970 | 88207578 | 81354790 | 90.61% | 83.57% |

**Table S2 27 DE-lncRNAs identified from twins**

| **Basic Information** | | | **case vs con (5BDC,5vs5)** | | | **case vs con (5BDC+4HCC, 5vs13)** | | |
| --- | --- | --- | --- | --- | --- | --- | --- | --- |
| **ID** | **SYMBOL** | **Type** | **edgeR.logFC** | **edgeR.p.value** | **edgeR.adj.p.value** | **edgeR.logFC** | **edgeR.p.value** | **edgeR.adj.p.value** |
| ENSG00000261534 | AL596244.1 | lncRNA | -1.19494567 | 0.029686297 | 1 | -1.53216736 | 0.019052923 | 0.997363651 |
| ENSG00000227544 | AC018647.1 | lncRNA | -1.28612305 | 0.038484974 | 1 | -1.745056315 | 0.023257674 | 0.997363651 |
| ENSG00000261286 | ATP2C2-AS1 | lncRNA | 1.233657436 | 0.008570463 | 1 | 1.305874689 | 0.049251833 | 0.997363651 |
| ENSG00000260526 | AP1AR-DT | lncRNA | 0.939918171 | 0.011253501 | 1 | 0.832909939 | 0.049407303 | 0.997363651 |
| ENSG00000235321 | AC007556.1 | lncRNA | 1.18580308 | 0.0044498 | 1 | 1.82158723 | 0.004154362 | 0.997363651 |
| ENSG00000230470 | AL078645.1 | lncRNA | -0.89088737 | 0.035201873 | 1 | -1.160835244 | 0.01150613 | 0.997363651 |
| ENSG00000180846 | CSNK1G2-AS1 | lncRNA | -0.62152689 | 0.009857755 | 1 | -0.74067504 | 0.014104688 | 0.997363651 |
| ENSG00000226310 | AL022157.1 | lncRNA | 1.438796594 | 0.026984533 | 1 | 1.584206524 | 0.014239409 | 0.997363651 |
| ENSG00000267028 | TCF4-AS1 | lncRNA | 1.164696691 | 0.025777987 | 1 | 2.121410259 | 0.014447821 | 0.997363651 |
| ENSG00000249684 | AC106795.2 | lncRNA | -0.78308143 | 0.043072273 | 1 | -1.169819628 | 0.016602548 | 0.997363651 |
| ENSG00000223949 | ROR1-AS1 | lncRNA | -3.11778828 | 0.013380118 | 1 | -2.938349902 | 0.020056628 | 0.997363651 |
| ENSG00000231019 | LINC00373 | lncRNA | -2.21074142 | 0.032370022 | 1 | -2.628649453 | 0.021144214 | 0.997363651 |
| ENSG00000231057 | AC096637.2 | lncRNA | -2.64438986 | 0.018618603 | 1 | -3.322130412 | 0.021259424 | 0.997363651 |
| ENSG00000214145 | LINC00887 | lncRNA | -0.64372564 | 0.007100635 | 1 | -0.798109463 | 0.025163931 | 0.997363651 |
| ENSG00000259319 | AF111167.2 | lncRNA | 0.649802452 | 0.036341506 | 1 | 1.310209252 | 0.027721295 | 0.997363651 |
| ENSG00000233303 | XXYLT1-AS1 | lncRNA | -2.11777884 | 0.026156093 | 1 | -2.164110926 | 0.030209666 | 0.997363651 |
| ENSG00000258655 | ARHGAP5-AS1 | lncRNA | 1.427365683 | 0.03405032 | 1 | 1.906125909 | 0.030258511 | 0.997363651 |
| ENSG00000237011 | AL357793.2 | lncRNA | 0.722954419 | 0.03923393 | 1 | 1.10774831 | 0.031819692 | 0.997363651 |
| ENSG00000227297 | AL035541.1 | lncRNA | 1.339290985 | 0.029626474 | 1 | 2.314628041 | 0.032327324 | 0.997363651 |
| ENSG00000221953 | C1orf229 | lncRNA | -2.27371699 | 0.006170302 | 1 | -2.799053586 | 0.034080578 | 0.997363651 |
| ENSG00000239705 | AL354710.2 | lncRNA | 1.084168406 | 0.028709411 | 1 | 1.239994073 | 0.034998185 | 0.997363651 |
| ENSG00000264031 | ABHD15-AS1 | lncRNA | -1.52459762 | 0.000159705 | 0.714486369 | -1.169063358 | 0.036743561 | 0.997363651 |
| ENSG00000261449 | AC103724.3 | lncRNA | 0.839747101 | 0.032365606 | 1 | 1.161751267 | 0.037748617 | 0.997363651 |
| ENSG00000226842 | AL158834.1 | lncRNA | -1.08221343 | 0.035100403 | 1 | -0.975641754 | 0.042228327 | 0.997363651 |
| ENSG00000197210 | AP000550.1 | lncRNA | -1.58092827 | 0.044710057 | 1 | -1.599018025 | 0.045023965 | 0.997363651 |
| ENSG00000258875 | AL135818.1 | lncRNA | 0.675491816 | 0.015558705 | 1 | 0.605688985 | 0.045357649 | 0.997363651 |
| ENSG00000258424 | AL512791.1 | lncRNA | 0.590334096 | 0.015411518 | 1 | 0.661855974 | 0.04993195 | 0.997363651 |

**Table S3 Primers and probes employed in this study**

| **ChIP** |  |  |
| --- | --- | --- |
| CHIP-F | GCACGACACGCTCTCTATCA |  |
| CHIP-R | GCAGGACGCCCCTCTTTTAT |  |
|  |  |  |
| **qPCR** |  |  |
| AP1AR-DT-F | AGACGGTTCAATTCCTGCGT |  |
| AP1AR-DT-R | CTAAGCACACCGTGCAGAGA |  |
|  |  |  |
| NEGR1-F | TAAATCTGGCACCGTGACCC |  |
| NEGR1-R | GGGCTGTACTTGGAGGGTT |  |
|  |  |  |
| NRF1-F | GAATTGCCAACCACGGTCAC |  |
| NRF1-R | TGGTCATCTCACCTCCCTGT |  |
|  |  |  |
| Gapdh-F | AGGGCTGCTTTTAACTCTGGT |  |
| Gapdh-R | CCCCACTTGATTTTGGAGGGA |  |
|  |  |  |
| β-actin-F | GGGAAATCGTGCGTGACATT |  |
| β-actin-R | GGAGTTGAAGGTAGTTTCGTG |  |
|  |  |  |
| **Plasmid cloning** |  |  |
| pGL4.18-NEGR1-Promotor-F | CCGCTCGAGTTACATAACTGGACCGCCTGG |  |
| pGL4.18-NEGR1-Promotor-R | CCCAAGCTTGCCCGTATGGATTGCAGGAC |  |
|  |  |  |
| pCDNA3.1+NRF1-F | CTAGCTAGCATGGAGGAACACGGAGTGAC |  |
| pCDNA3.1+NRF1-R | CCGCTCGAGTCACTGTTCCAATGTCACCACC |  |
|  |  |  |
| pCDH-AP1AR-DT-F | CGGGAATTCCCTGGTTCAGCCGCCTCTGC |  |
| pCDH-AP1AR-DT-R | CGGGATCCTTTTGATATTTACCATCCAGCCTTTATTTTCAAG | |
|  |  |  |
| **ChIRP** |  |  |
| probe1 | GTCAAGTCTATAGCTGCATT |  |
| probe2 | TGTATTGCTTCTGAGGATCA |  |
| probe3 | CCGAAACTGCAATGAGAGAG |  |
| probe4 | ACAAAGTTCGAAGGCTGAGC |  |
| probe5 | TGGTGCGTTCAAAGTAACCT |  |
| probe6 | GATGTGTCCAGTCCATGAGA |  |
