# Supplementary material for: Gain of bipolar disorder-related lncRNA AP1AR-DT in mice induces depressive and anxiety-like behaviors by reducing Negr1-mediated excitatory synaptic transmission
Source: BMC Med. 2024 Nov 18;22:543. doi: 10.1186/s12916-024-03725-0 (PMC11575081; doi:10.1186/s12916-024-03725-0)
Supplement: Supplementary file 2 — Additional file 2: Figs. S1-S4. Fig S1- [The information of AP1AR-DT]. Fig S2- [Behavioral results of AP1AR-DT overexpression in the three-chamber test, Barnes maze test and sucrose preference test]. Fig S3- [The location of binding sequence of AP1AR-DT to NEGR1 promoter region and every primer sequence on promoter region]. Fig S4- [Representative traces of pair-pulse stimulation responses and pair-pulse ratios plotted against interstimulus intervals]. [file 12916_2024_3725_MOESM2_ESM.docx]

**Supplementary Figures**

**
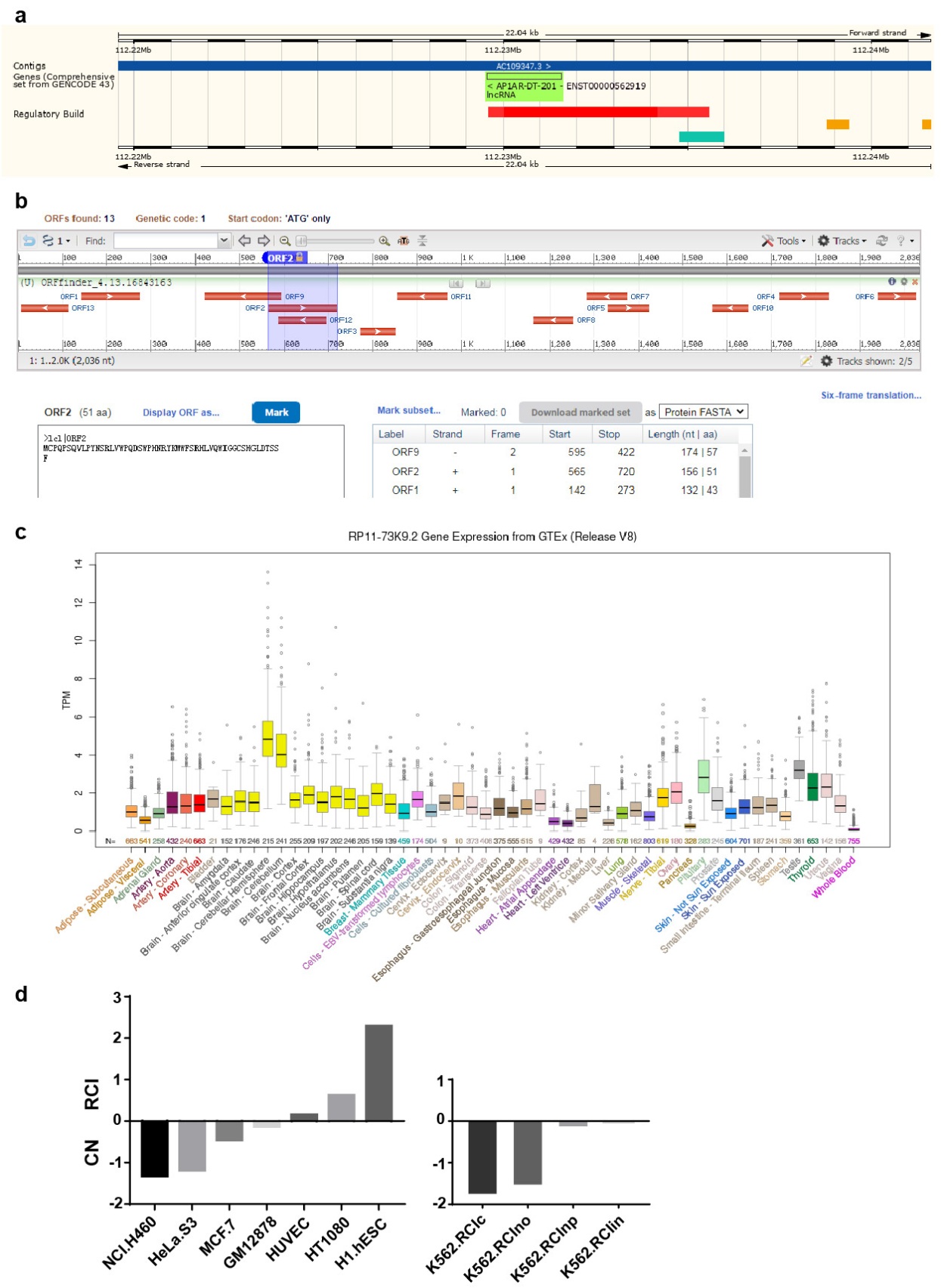
**

**Figure S1.** (**a**) Annotation of lncRNA *AP1AR-DT* shown in the Ensembl database. (**b**) *In-silico* coding potential analysis of *AP1AR-DR* with the open reading frame (ORF) prediction tool ORFfinder. (**c**) Tissue expression patterns of *AP1AR-DT* from GTEx (https://www.gtexportal.org/home/index.html) data sets. (**d**) The subcellular localization of *AP1AR-DT* was extraordinarily nuclear rather than cytoplasmic, as determined RNA-seq data from HUVECs from the lncATLAS dataset (<http://lncatlas.crg.eu>).

**
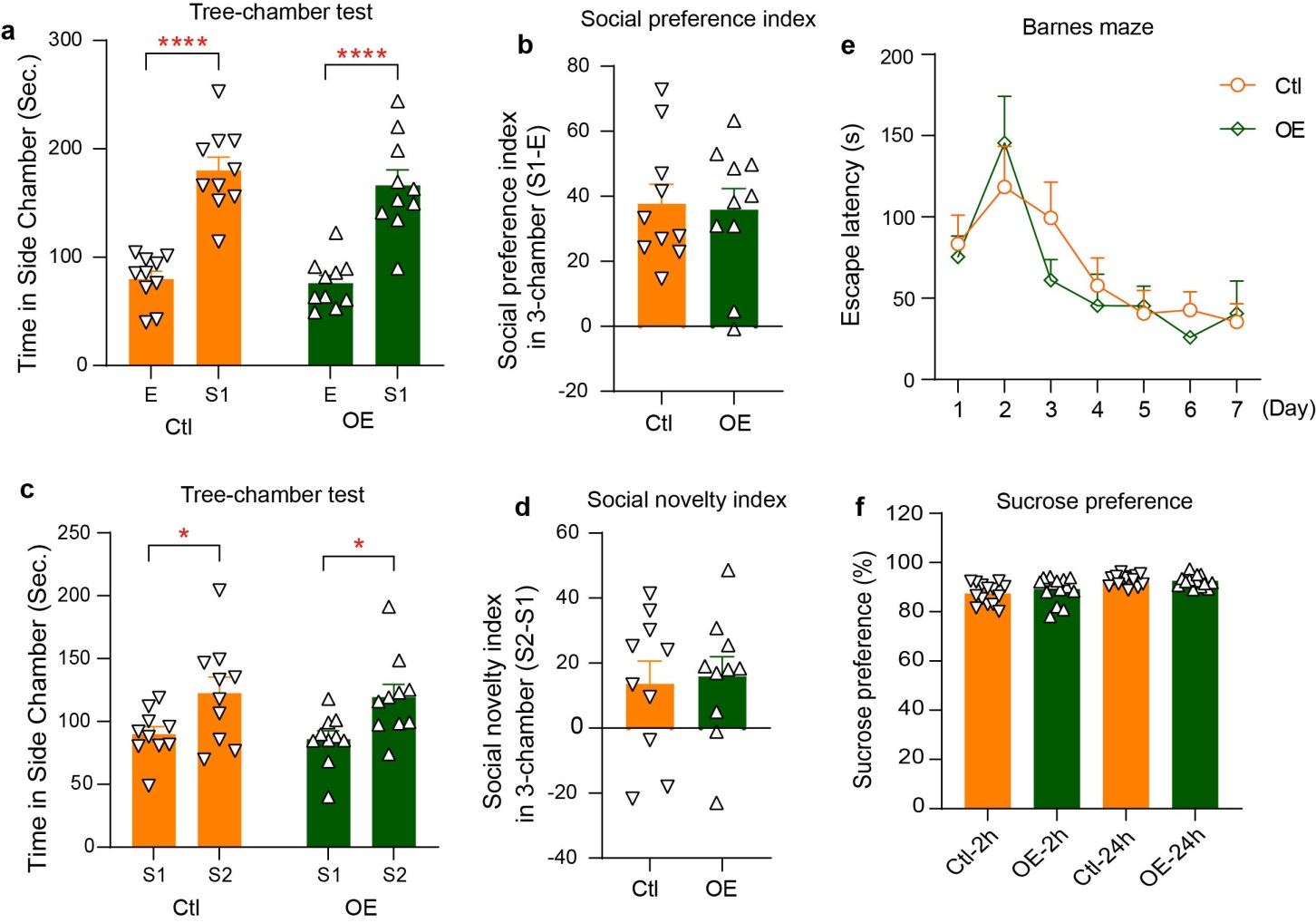
**

**Figure S2.** Behavioral results of *AP1AR-DT* overexpression in the three-chamber test **(a-d),** in the Barnes maze test (**e**) and in the sucrose preference test (**f**).

**
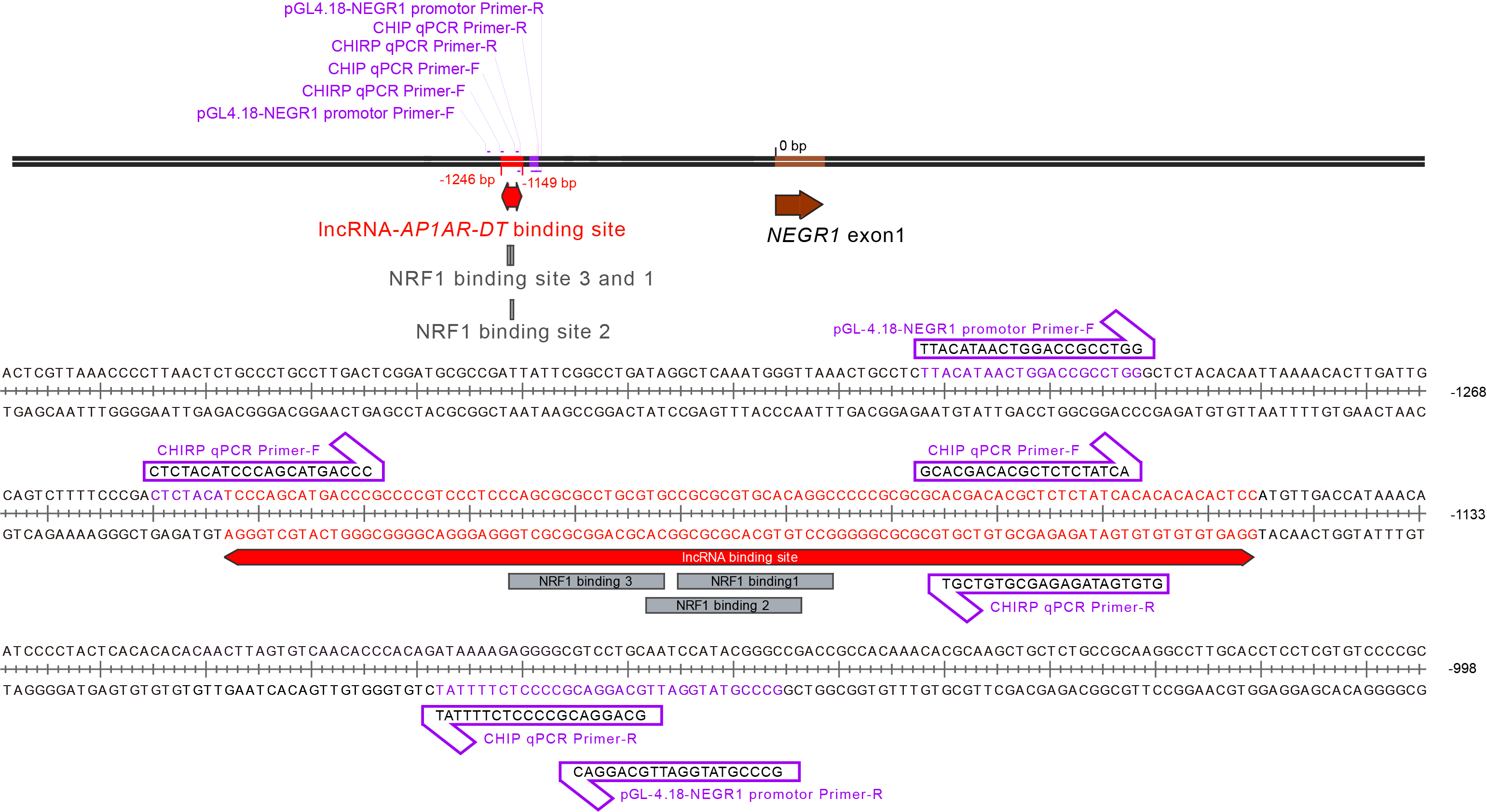
**

**Figure S3. The location of binding sequence of lncRNA-*AP1AR-DT* to *NEGR1* promoter region and each primer sequence on promoter region.**

**
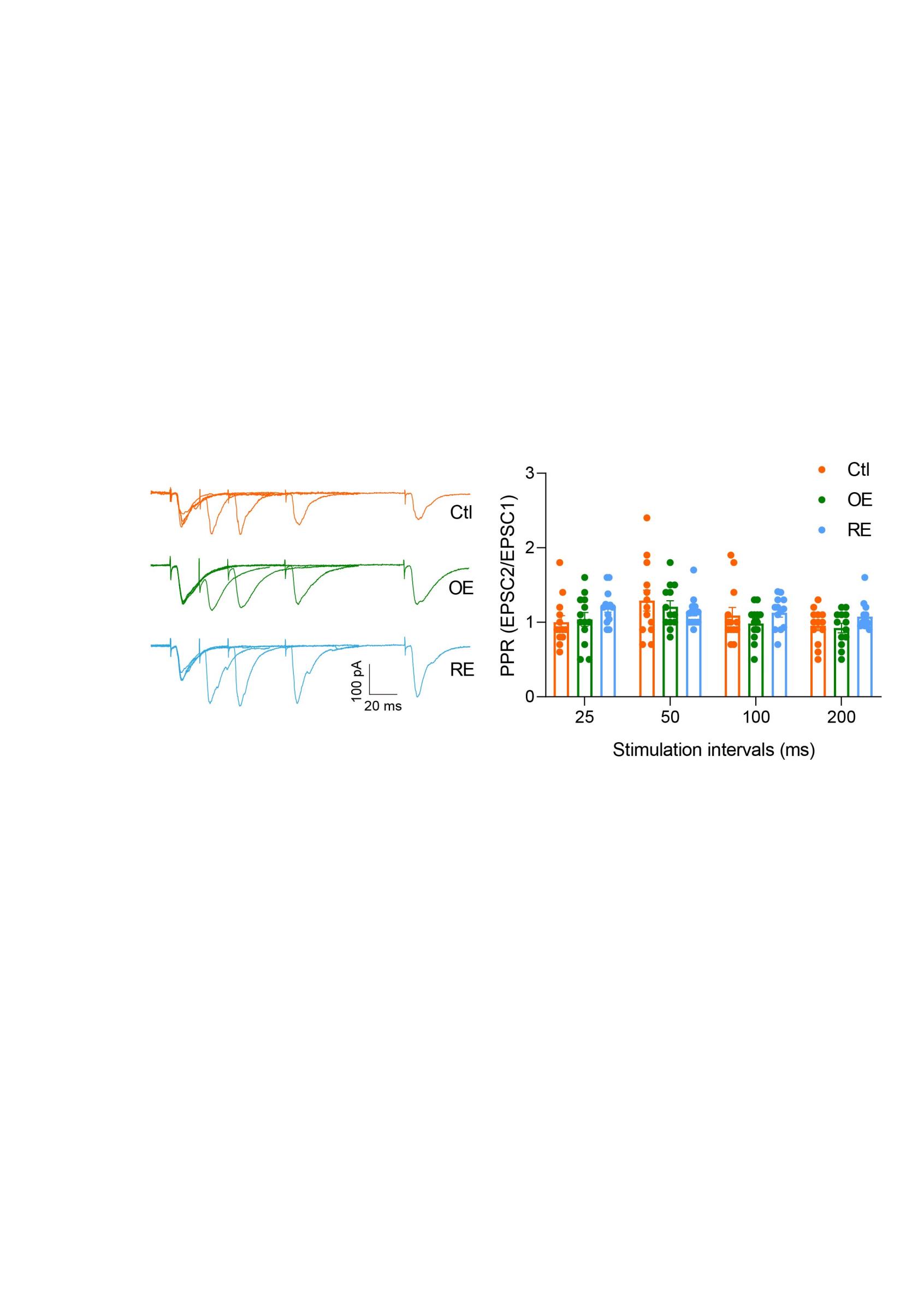
**

**Figure S4** Representative traces of pair-pulse stimulation responses (left) and pair-pulse ratios (PPRs) plotted against interstimulus intervals (right) show that PPRs remained unchanged among three groups. n = 13 cells from four Ctl mice, n = 18 cells from three OE mice, and n = 12 cells from three RE mice. A two-tailed t-test was used for comparisons between the two indicated groups (**P* < 0.05, ***P* < 0.01, ****P* < 0.001, and *****P* < 0.0001).
