# Supplementary material for: Gain of bipolar disorder-related lncRNA AP1AR-DT in mice induces depressive and anxiety-like behaviors by reducing Negr1-mediated excitatory synaptic transmission
Source: BMC Med. 2024 Nov 18;22:543. doi: 10.1186/s12916-024-03725-0 (PMC11575081; doi:10.1186/s12916-024-03725-0)
Supplement: Supplementary file 3 — Additional file 3. Original images for Figs. 4B, 4C, 5E and 6E. [file 12916_2024_3725_MOESM3_ESM.docx]

**
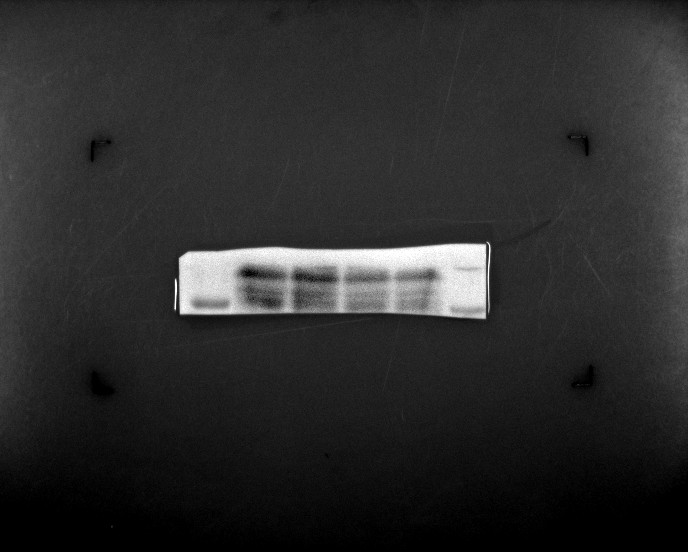
**

1. **Original image for Negr1 blotting in Figure 4B**

**
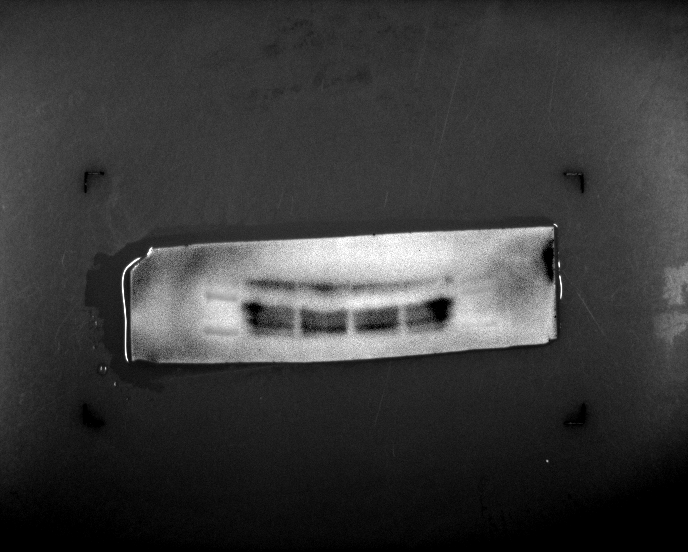
**

1. **Original image for b-tubulin blotting in Figure 4B**

**
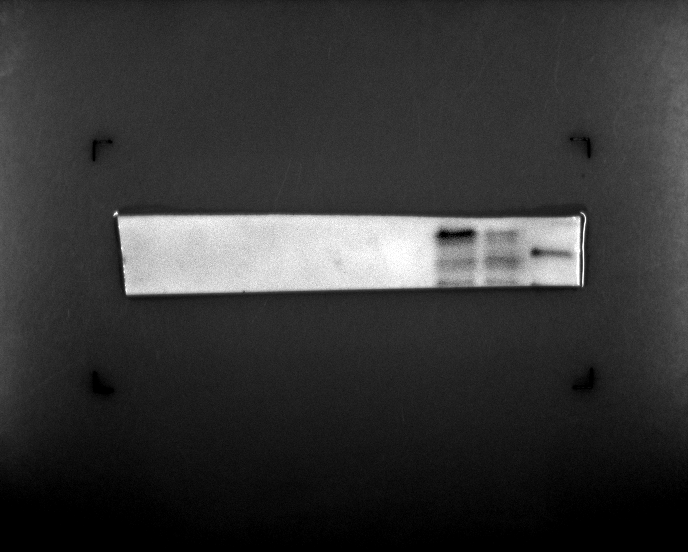
**

1. **Original image for NEGR1 blotting in Figure 4C**

**
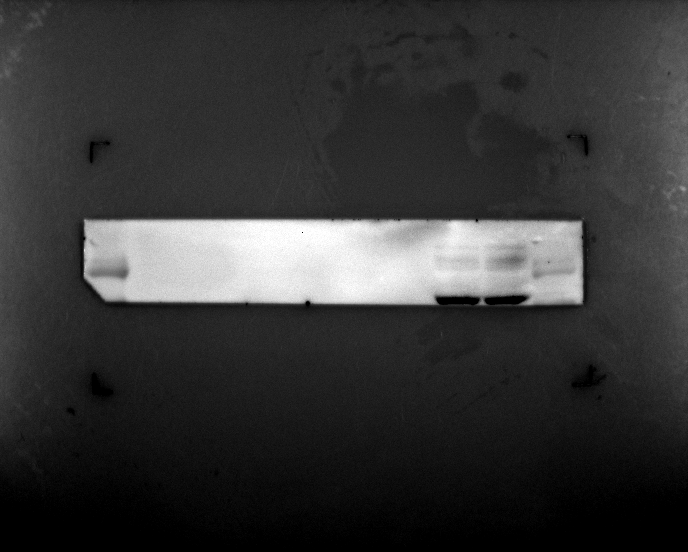
**

1. **Original image for b-actin blotting in Figure 4C**

**
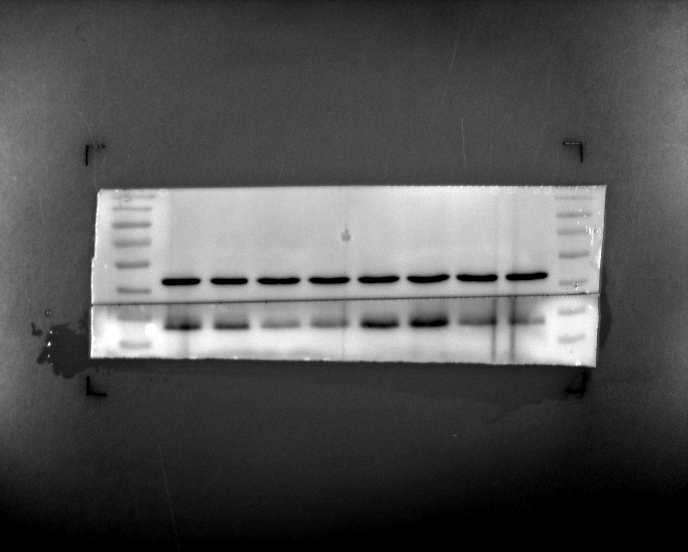
**

1. **Original image for Figure 5E**

**
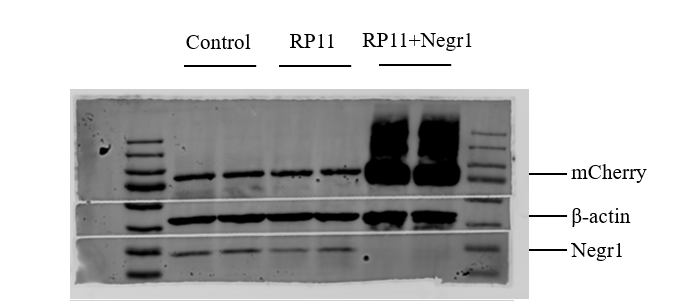
**

1. **Original image for Figure 6E**
